# Decoding short-term fertility intentions: exploring the nexus of gender equality and societal factors in a comparative EU gender regimes analysis

**DOI:** 10.3389/fsoc.2025.1651929

**Published:** 2025-10-16

**Authors:** Alba-María Aragón-Morales, Antonia-María Ruiz-Jiménez

**Affiliations:** Pablo de Olavide University, Seville, Spain

**Keywords:** fertility intentions, gender regimes, gender equality, comparative analysis, classification and regression trees (CART), family policies

## Abstract

**Background:**

Persistently low fertility in the European Union has drawn attention to the gap between desired and intended fertility, often linked to enduring gender inequalities. Clarifying how individual, partner, and contextual factors jointly shape short-term fertility intentions can inform policy across diverse gender regimes. Objective: To examine short-term fertility intentions among partnered individuals in Finland, Germany, and Spain, representing Scandinavian, Continental, and Mediterranean gender regimes, respectively.

**Methods:**

We use harmonized data from the Generations and Gender Survey (GGS) and Spain’s National Institute of Statistics (INE). We apply Classification and Regression Trees (CART) to capture non-linear interactions among individual, partner, and contextual factors (including employment status, caregiving responsibilities, and gender values), and to identify profiles associated with higher vs. lower short-term intentions to have a child.

**Results:**

Family size, caregiving burdens, and economic stability emerge as central determinants of fertility intentions, with marked gendered and contextual differences across countries. Patterns are particularly pronounced among individuals with no children or one child, where combinations of stable employment and lower caregiving loads align with higher intentions, while economic insecurity and heavier (gendered) care burdens depress intentions. CART uncovers country-specific thresholds and configurations consistent with each gender regime.

**Conclusion:**

Short-term fertility intentions reflect unmet gender-mediated needs and serve as an early indicator of latent potential for social and political mobilization. Our findings highlight the influence of gender regimes on reproductive decision-making and support policies that address structural inequalities, especially in employment and care, to enable the realization of reproductive desires across heterogeneous socioeconomic contexts.

## 1 Introduction

In recent decades, the combination of declining fertility rates and increased life expectancy has led to what is often termed “demographic aging.” While this phenomenon is sometimes viewed negatively, it can also be seen as a sign of progress in equality and social well-being within developed democracies, resulting in longer and higher-quality lives (Pérez, 2020). Nevertheless, these positive trends also create new pressures on welfare systems. The demographic transformation poses significant challenges for the sustainability of welfare states, especially regarding public services such as pensions, healthcare, and education. At the same time, changing family structures require more nuanced family policies that address family diversity, caregiver support, work-life balance, and co-responsibility (Billingsley and Ferrarini, 2014).

The pressing issue of low fertility in many European Union countries has captured the attention of national and supranational entities, as well as the media, prompting extensive research into its complex drivers. Previous studies have underscored a disparity between desired (ideal) fertility and actual short-term fertility intentions in European states (Dey and Wasoff, 2010; Beaujouan and Berghammer, 2019). Feminist literature, for instance, through concepts like the ‘stalled revolution’ (Goldscheider et al., 2015) or analyses of the ‘second shift’ (Hochschild and Machung, 1989), often attributes this gap to persistent gender inequalities across realms crucial for childrearing. These include disparities in the labor market, challenges in work-life balance, unequal access to economic resources, and the inequitable distribution of caregiving responsibilities. Building on this feminist scholarship, comparative research has increasingly examined how these gender inequalities manifest differently across national contexts, with studies documenting significant variation in the relationship between gender equality and fertility across European countries (Neyer et al., 2013; McDonald, 2013). Research has shown that contextual factors—including institutional arrangements, family policies, and cultural norms—interact with individual-level gender dynamics to shape reproductive decisions in complex ways (Pfau-Effinger, 2017; Brinton et al., 2018).

This body of comparative research has highlighted the importance of understanding fertility intentions within broader frameworks that capture systematic differences in gender arrangements across societies. To address these complexities, this study draws on the concept of “gender regimes” from feminist research. Gender regimes refer to the structures and relationships that shape gender equality and family systems (Giordano, 2019). They play a crucial role in influencing family norms related to parenthood, motherhood, and childrearing, and thus affect fertility intentions (Mönkediek and Bras, 2017). Despite the importance of this framework, there is still a research gap regarding how different levels of gender equality within national contexts systematically influence short-term fertility intentions.

To investigate these dynamics, this study focuses on Finland, Germany, and Spain. These countries represent three distinct gender regimes in Europe: the Scandinavian model (Finland, characterized by high gender equality and comprehensive welfare); the Continental model (Germany, often seen as a model transitioning from a strong male breadwinner system toward one with greater emphasis on dual earners and shared care, albeit with persistent traditional elements and moderate equality); and the Mediterranean model (Spain, traditionally marked by strong family ties and notable gender inequalities) (Giordano, 2019). Their contrasting approaches to gender equality, family policies, and societal norms provide a rich comparative framework. These regimes are theoretically relevant for understanding fertility intentions, as they institutionalize different approaches to the work-family intersections; gender equality in both public and private spheres; and the level of state support for families—all crucial elements that influence reproductive decision-making (Dommermuth et al., 2011, 2015).

This research explores how these contextual factors, in conjunction with individual characteristics, shape short-term fertility intentions. We focus on short-term fertility intentions because they are proximal to behavior and capture the perceived feasibility of childbearing under current constraints (Ajzen, 1991; Ajzen and Fishbein, 1973), making them highly sensitive to the structural and cultural mechanisms we study. Specifically, this study addresses the following questions: (1) To what extent do individual and partner factors (such as employment, resources, caregiving distribution, and gender attitudes) contribute to variations in short-term fertility intentions? (2) How do these determinants differ between men and women in distinct gender regimes? To answer these questions, we developed predictive models using Classification and Regression Trees (hereinafter, CART) based on quantitative analysis of data from the Generations and Gender Survey (GGS) and the Spanish Fertility Survey (INE). CART is a non-parametric, interpretable machine-learning method that uncovers interaction-based decision rules and threshold effects—precisely the kind of complex, contingent patterns expected when gendered resources, care arrangements, and attitudes jointly shape short-term fertility intentions.

Furthermore, this paper frames short-term fertility decisions within the broader context of gender-based needs. The desire to have children—or the decision to postpone or forego it—is intrinsically linked to gendered expectations, career trajectories, and the unequal distribution of care work. When structural conditions (such as economic instability, lack of affordable childcare, or precarious employment) hinder these personal life projects, the resulting individual decisions can be interpreted as a form of passive mobilization. Collectively, these patterns signal a gap between personal aspirations and societal support, highlighting the mobilization potential inherent in these fundamental, gendered needs.

The remainder of this article is structured as follows: We first delve into theoretical conceptualizations of “gender regimes” and review previous research on factors influencing fertility intentions in Europe, which informs our variable selection. Subsequently, we present the country-specific results derived from our CART analyses. The discussion then synthesizes these findings, exploring cross-cutting themes such as potential regime evolution and hybridization, the varying salience of the analytical dimensions across contexts, and considers their policy implications. Finally, we reflect on the study’s contributions, acknowledge its limitations, and suggest promising avenues for future research, aiming to enhance the ongoing scholarly agenda on fertility in Europe.

## 2 Gender regimes and fertility: Does gender regime matter?

In recent decades, most developed countries have witnessed profound transformations in gender roles and attitudes. Two processes have unfolded at different speeds. First, practices in the public sphere have shifted markedly—women’s educational attainment and labor force participation have risen substantially. Second, attitudinal change toward gender egalitarianism has advanced, though unevenly across contexts. By contrast, practices in the private sphere—especially the division of care and housework—have often lagged behind. While some European nations, primarily those in Scandinavia, are advancing toward “gender egalitarianism” in both public and private spheres, others exhibit significant shifts in the former without corresponding adjustments in the latter. This situation has led to what some scholars describe as “incomplete” or “stalled” gender revolutions (Friedman, 2015; Sullivan et al., 2018). This “stall” is particularly evident when women’s notable increases in educational attainment and labor market participation (a public sphere revolution) are not matched by a transformation in traditional expectations regarding their primary responsibility for childcare and housework (a lack of revolution in the private sphere). The concept of the “stalled revolution” is especially pertinent when analyzing fertility intentions within different gender regimes, as the intensified tension between work and family life stemming from this mismatch can influence the perceived feasibility and desirability of having children (Goldscheider et al., 2015; Riederer et al., 2019). Therefore, this study considers how the characteristics of Continental and Mediterranean gender regimes, often associated with this “stalled revolution,” might shape the landscape of fertility intentions, particularly in comparison with Scandinavian regimes where gender equality and work-family balance are often more advanced.

Before proceeding with our theoretical framework, it is essential to clarify the key gender-related terminology used throughout this study, given the centrality of these concepts to our analysis. Gender equality refers to the formal and substantive equal treatment of individuals regardless of gender, encompassing equal access to opportunities, resources, and rights in both public and private spheres (Walby, 2020). This includes measurable indicators such as labor force participation rates, wage gaps, and political representation. Gender equity, while related, emphasizes fairness and justice in treatment, recognizing that achieving equality may require different approaches for different groups to address historical disadvantages and structural barriers (Goldscheider et al., 2015). In the context of fertility research, equity often refers to fair distribution of caregiving responsibilities and domestic labor within couples, which may not necessarily mean a 50/50 split but rather a division perceived as just by both partners. Egalitarianism represents the broader ideological commitment to equality as a social value, encompassing both attitudes and practices that promote equal treatment and opportunities across gender lines (Raybould and Sear, 2021). Throughout this paper, we use “gender equality” as our primary umbrella term when referring to the broader societal and institutional arrangements, while employing “equity” specifically when discussing fairness in domestic arrangements and “egalitarianism” when referring to value orientations and attitudes.

### 2.1 Defining gender regimes: ranking European countries

This section delves into the complex interplay between gender dynamics and short-term fertility intentions. While prior research indicates a positive correlation between broader societal gender equality and childbearing plans, measurement approaches in earlier studies show important limitations. McDonald (2013) emphasizes institutional indicators such as women’s labor-force participation and educational attainment, and Fanelli and Profeta (2021) examine fathers’ involvement using time-use data and policy indicators like parental-leave availability. However, these macro-level indicators often fail to capture the micro-level dynamics of gender relations within couples and households. This gap is consequential: it overlooks how couples negotiate and reproduce gender roles in practice, which can help explain inconsistent findings about the gender-equality–fertility relationship across contexts.

Walby’s theorisation of “gender regimes” helps bridge this micro–macro divide by treating gender inequality as a system of interdependent structures — including state policy, market relations, household organization, culture, sexuality and violence — that are reproduced through everyday practices and normative agreements (the “gender contract”) between partners. From Walby’s (2000, 2004, 2009) perspective, institutional equality (for example, formal policies or labor-market indicators) is a necessary but not sufficient condition for egalitarian family life because the lived division of labor and normative expectations within households mediate how institutions actually affect reproductive decision-making. Thus, a more nuanced understanding requires explicit attention to the gender contract — the informal, negotiated set of expectations that shapes how men and women allocate paid and unpaid work, and how they perceive financial provision and caregiving (Pfau-Effinger, 1998; Lutz, 2011).

Building on this combined perspective, Giordano (2019) operationalizes a two-dimensional framework that evaluates both institutional/structural indicators of gender equality and the prevailing gender contract (attitudinal and practice-based measured). Using principal-component and cluster analyses across 21 European countries data, Giordano identifies distinct regime types that capture combinations of policy context and household-level norms. This bidimensional approach maps closely onto Walby’s argument that regimes are best understood as the interaction of macro structures and micro practices, and it therefore provides a useful empirical tool for studying fertility intentions in comparative perspective.

Giordano’s typology yields three models that are analytically useful for our comparative design. The first, the Scandinavian Regime (e.g., Finland, Sweden, and Denmark), is characterized by a “modern gender contract,” high levels of gender equality, and social progressivism, aligning with Esping-Andersen’s social-democratic welfare model (Esping-Andersen, 1999). The second, the Continental Regime (e.g., the UK, Germany, and France), features a “gender contract in transition” and varying degrees of gender equality across different domains, representing a midpoint in terms of gender traditionalism. Finally, the Mediterranean Regime (Southern and Eastern European countries, e.g., Spain) presents a “traditional gender contract,” greater gender inequality (particularly in the labor market, purchasing power, and caregiving), and conservative gender-family relations. Although Esping-Andersen’s original typology did not differentiate between Continental and Mediterranean countries, Giordano’s classification identifies notable gender-based differences between these latter two regimes.

**Figure 1:**
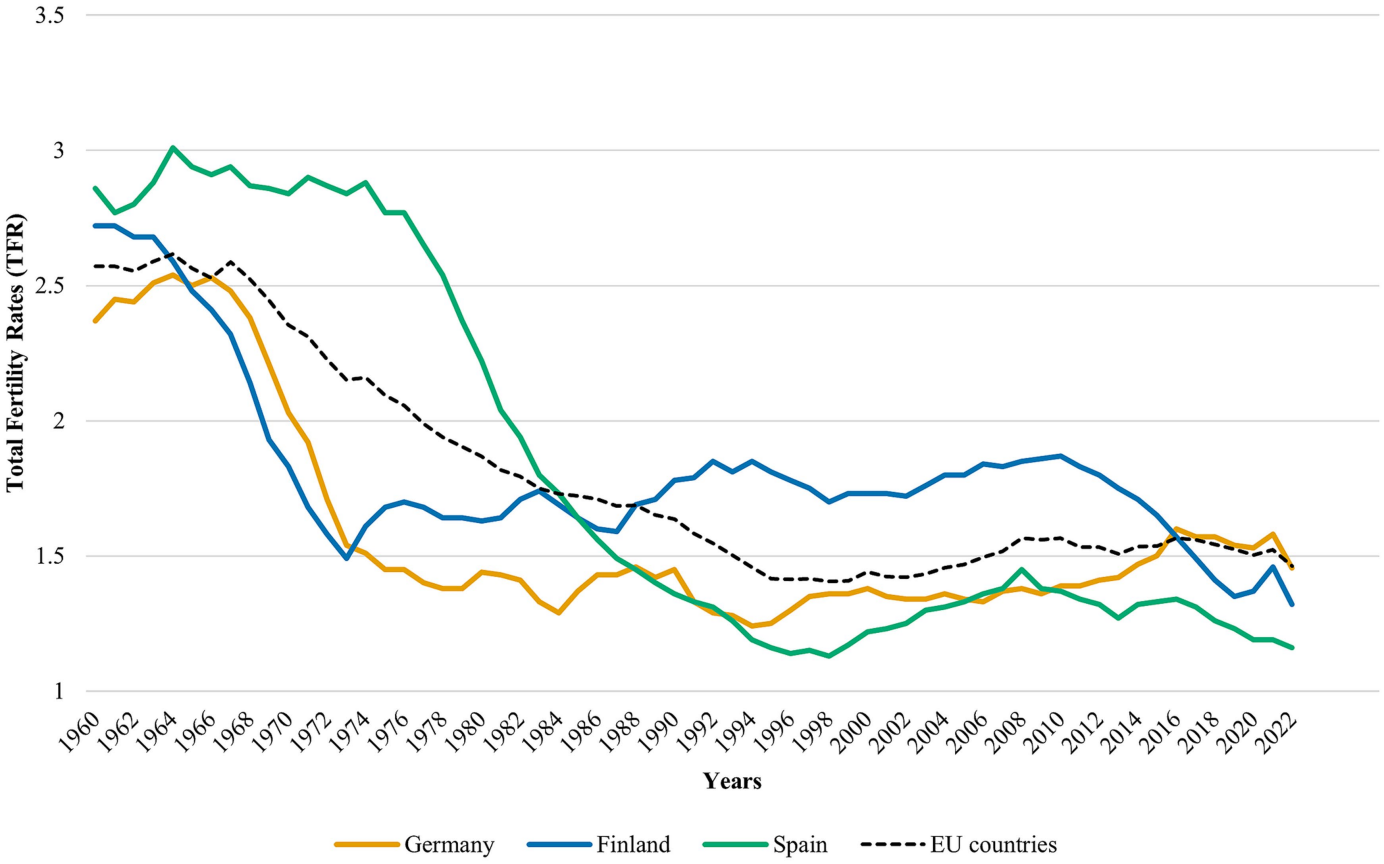
Evolution of total fertility rates in Finland, Germany, and Spain (1960–2022). Source: own elaboration based on World Bank data: fertility rates in Finland, Germany, Spain, and EU countries average.

While Giordano’s (2019) typology is widely utilized and provides a robust framework for our comparative analysis, it is worth noting that the scholarship on gender regimes is dynamic, with ongoing discussions regarding the precise boundaries of these categories or the inclusion of additional dimensions (Sainsbury, 1999; Walby, 2020). Nevertheless, the tripartite distinction adopted here offers a strong foundation for exploring variations in fertility intentions across these national contexts. For our study, we selected Finland, Germany, and Spain as representative cases, considering the significant transformations in their fertility rates (Figure 1), which reflect socioeconomic, policy, and cultural shifts.

Finland epitomizes the Scandinavian regime, and provides an interesting case study due to its gender equality performance (ranked 2nd globally in the Global Gender Gap Index 2024) and comprehensive family policies, including generous parental leave (up to 164 days that can be shared between parents) and universal childcare access (World Economic Forum, 2024; OECD, 2023). This combination of high gender equality and supportive family infrastructure makes Finland particularly relevant for examining how gender regimes influence fertility intentions. Following the post-war baby boom, Finland’s fertility rates declined significantly through the 1960s, reaching a historic low in the early 1970s as female workforce participation increased. This trend began to stabilize and reverse in subsequent decades, a shift largely attributed to a series of landmark family-friendly policies designed to address this challenge (Dioikitopoulos and Varvarigos, 2023). Key among these were the Act on Children’s Daycare of 1973, which established a universal right to public childcare, and the introduction of a home care allowance in 1985, offering parents a choice between subsidized care and staying at home. These measures, alongside the continuous expansion of parental leave schemes throughout the 1970s and 1980s, were designed to facilitate a dual-earner model supported by flexible working hours and substantial parental leaves (Golovina et al., 2024). This robust government support for families reduces the economic burden of childrearing, promoting work-life integration and gender parity.

Germany, as a Continental regime, is characterized by moderate gender equality and a selective welfare state. Its complex fertility history saw West Germany’s rates drop in the mid-1960s, while East Germany’s remained higher until the 1990 reunification triggered a sharp decline (Möhring and Weiland, 2021). In response to persistently low rates, a major policy shift in the mid-2000s introduced the earnings-related Parental Benefit (*Elterngeld*) in 2007 and expanded childcare access from 2013. Despite these reforms aimed at promoting parity, traditional roles still influence family life, and the labor market remains a hybrid of dual-earner households and the traditional model, with many women opting for part-time work post-childbirth (Kuhnt and Trappe, 2016; Leitner, 2016). Consequently, work-life balance policies are less comprehensive than in Scandinavian countries, with support often relying on family networks and employer policies (Trappe et al., 2015).

Spain embodies the Mediterranean regime, where traditional gender roles are more pronounced, and progress toward gender equality has been slower. Fertility, which was high in the 1960s and 1970s, fell sharply in the 1980s and 1990s with the mass entry of women into the workforce (Sobotka, 2017). Economic instability and changing values maintained a downward fertility trend until 2020 (Bueno and Brinton, 2019). The Spanish welfare state is less extensive, with limited state support, and childcare and parental leave services are less generous than in Finland or Germany. The male bread-winner model prevails, with many women assuming primary domestic responsibilities. Limited work-life balance policies (Suero, 2023) make it challenging to combine work and family, leading to a high reliance on extended family networks for childcare. These cases illustrate how distinct regimes manifest through differentiated approaches to equality, welfare, family support, and work-life balance, reflecting cultural and policy differences that shape lived experiences.

### 2.2 Explaining short-term fertility intentions: background for constructing predictive models

This section establishes the conceptual and operational groundwork for constructing predictive models of short-term fertility intentions. Building robust and insightful models in this domain requires a theoretically grounded and empirically validated approach to selecting and organizing explanatory factors. Therefore, we will first discuss the operationalization of our dependent variable, short-term fertility intentions. Following this, we will systematically review the key dimensions and specific variables—spanning individual, couple, and contextual levels—that prior research has identified as significant predictors. This review will not only justify the inclusion of these factors in our models but also structure the subsequent discussion around the core dimensions that underpin our analytical strategy, aiming to elucidate how different elements contribute to the decision-making process regarding near-future childbearing.

The fertility literature distinguishes between several related but conceptually distinct measures of reproductive preferences. Fertility ideals represent individuals’ abstract preferences about family size under ideal circumstances, often reflecting societal norms and cultural expectations about the “perfect” family (Philipov and Berghammer, 2007). These ideals are typically measured through questions such as “What do you think is the ideal number of children for a family like yours?” Fertility desires capture personal wishes about childbearing, usually assessed through questions about wanting additional children, but without specific temporal constraints or consideration of practical constraints. Fertility intentions, in contrast, represent concrete plans to have children within a specified timeframe, incorporating both personal preferences and perceived feasibility given current circumstances (Ajzen, 1991).

This study examines short-term fertility intentions rather than fertility ideals, desires, or actual fertility outcomes for several key reasons. Unlike fertility ideals (abstract preferences about family size under ideal circumstances) or desires (personal wishes without temporal constraints), intentions represent concrete plans that incorporate both preferences and perceived feasibility given current circumstances (Ajzen, 1991). Intentions are superior predictors of actual fertility behavior, particularly for partnered individuals over a three-year horizon (Brinton et al., 2018; Duvander et al., 2020), and are more sensitive to contemporary gender regime factors than either abstract ideals or aggregate fertility rates.

Most importantly for our theoretical framework, fertility intentions capture the intersection between personal desires and structural constraints—precisely where gender regimes exert their influence. The Theory of Planned Behavior (TPB) conceptualizes intentions as motivational factors shaped by attitudes, subjective norms, and perceived behavioral control (Ajzen, 1991). This framework helps explain how gender regime characteristics—such as employment policies, childcare availability, and cultural norms about gender roles—translate into individual reproductive preferenc. By focusing on short-term intentions, we capture individuals’ real-time assessments of their capacity to realize reproductive goals within the constraints and opportunities of their specific gender regime context, making intentions the most appropriate measure for examining how employment conditions, economic resources, caregiving arrangements, and gender values interact across different institutional settings.

Having established the theoretical rationale for examining short-term fertility intentions, we now turn to the systematic review of factors that prior research has identified as significant predictors. This review serves a dual purpose: it justifies the inclusion of specific variables in our predictive models and structures our analytical strategy around four core dimensions that capture how gender regimes influence reproductive decision-making.

The debate persists on whether gender equality promotes childbearing intentions, with empirical evidence varying according to the equality indicators used, the samples studied, and the country’s gender regime studied (Neyer et al., 2013). To address this complexity, our analysis operationalizes gender regime influences through four key dimensions: economic resources, employment conditions, caregiving distribution, and gender values. Our four analytical dimensions derive directly from TPB and the gender regimes literature. Economic resources and employment conditions map onto perceived behavioral control; caregiving distribution speaks to perceived fairness and feasibility in the private sphere; gender and family attitudes capture both attitudes and internalized norms. Family policies cut across these dimensions by shaping labor market risks, care infrastructures, and normative expectations. Organizing predictors in this way ensures theoretical alignment and prepares the ground for CART to detect contingent, regime-specific interactions.

Regarding economic resources and employment, job security and sufficient resources may influence women’s short-term intentions, especially for childless or full-time employed women (Neyer and Rieck, 2009; Testa, 2012; Fahlén, 2013), an effect potentially more pronounced in Continental and Mediterranean regimes with less state support. Conversely, other studies have indicated that employed women report lower short-term fertility intentions, attributing this to higher career opportunity costs (Matysiak and Vignoli, 2008). This tension might be particularly acute in contexts where public policies and workplace cultures are not fully aligned with a dual-earner/dual-carer model, a situation common in Continental regimes.

In contrast to the complex and sometimes negative association between employment and fertility intentions observed for women, most studies have agreed that employed men exhibit higher short-term childbearing intentions or that employment has no discernible effect on their intentions. This phenomenon is often attributed to the persistence of “gender contracts” tied to the “male breadwinner” model, as well as men’s lesser involvement in caregiving responsibilities, which reduces the impact of children on their labor market situation compared to women (Won et al., 2023).

Furthermore, it is commonly assumed that greater gender equality in the division of household and caregiving responsibilities enhances short-term childbearing intentions for both sexes. However, research yields inconsistent results, contingent on the country studied women’s workload (whether from employment or the number of children) and the extent of fathers’ involvement (Aassve et al., 2015). The impact of caregiving distribution is likely highly sensitive to the gender regime; for instance, an unequal division might be a stronger deterrent for women’s fertility intentions in Scandinavian countries, with strong norms of paternal involvement, than in Mediterranean regimes, where traditional divisions may be more normative and alternative (family) support plays a role. The evidence suggests that the effect may vary depending on whether fathers are engaged in childcare or general housework and whether women are satisfied with the division of family labor, whether it is equal or not.

Goldscheider et al.’s (2015) “gender revolution” hypothesis predicts a U-shaped relationship between fertility and gender equity in Europe. Fertility would be higher with very low gender equity, decline to deficient levels at moderate equity (where women experience equity in public institutions but not in family institutions—the “stalled” phase with a “double burden,” potentially descriptive of some Continental regimes), and then increase as the gender revolution progresses toward equity in both spheres (as aspired to in Scandinavian regimes). Building on this, Raybould and Sear (2021) redefined the theory, suggesting that fertility tends to be moderate when there is a balanced (not necessarily equal, but perceived as fair) sharing of responsibilities. Fertility reaches its lowest point in societies with strict gender norms, where women engage in paid work that is misaligned with childcare. As societies adopt more flexible gender norms and men increase their share of domestic duties, fertility is expected to rise, a transition occurring at different paces and forms across regimes.

Regarding gender and family values, religiosity is examined in fertility research because it is strongly connected to gender and family attitudes that shape reproductive behavior. Some studies have considered religiosity to explain fertility intentions, finding significant effects, especially long-term, mediated by family support (Dantis et al., 2023). Vogl and Freese (2020) noted that individuals with conservative gender and family values (especially on abortion and same-sex marriage) tend to have higher fertility intentions. This influence might be more pronounced in Mediterranean regimes with a stronger historical weight for traditional family institutions and religiosity than in Scandinavian countries with more secularized orientations (Bein et al., 2021). Continental regimes may exhibit a broader spectrum of value orientations.

In summary, the literature reviewed in this section underscores that a comprehensive understanding of short-term fertility intentions necessitates an examination of multiple, interacting factors. The theoretical frameworks and empirical evidence consistently point toward the significance of individuals’ employment circumstances and their perceived financial situation as crucial preconditions. Furthermore, the perceived internal dynamics of couples, particularly the distribution of and satisfaction with domestic and caregiving responsibilities, emerge as pivotal. Finally, overarching family and gender attitudes held by individuals shape their aspirations and perceived feasibility of having children. These dimensions, as will be detailed in the subsequent operationalization of our variables, provide a structured approach to modeling fertility intentions across different gender regimes.

## 3 Materials and methods

This study employs a comparative approach to analyze short-term fertility intentions across three European countries—Finland, Germany, and Spain—representing distinct gender regimes. Using data from the Generations and Gender Survey (GGS) and the Spanish National Institute of Statistics (INE), classification and regression trees (CART) were used to identify and analyze the key factors influencing fertility intentions.

### 3.1 Data sources and sample selection

We examine the fertility intentions of women and men in three countries representative of Giordano’s (2019) gender regime models. Data for Finland and Germany come from the GGS 2020 (Round 2, Wave 1), fielded in 2020/2021, whereas Spain’s data were from the INE 2018 Fertility Survey, which includes comparable variables. Both surveys target the general population (18–59 years old) with representative samples: 3,388 for Finland, 22,048 for Germany, and 17,175 for Spain.

The unavailability of GGS data for Spain coupled with the notable absence of any country representing the Mediterranean gender regime within the GGS dataset, prompted the use of these two data sources. The INE Fertility Survey was selected for its comparable variables on fertility intentions and sociodemographic characteristics. The Instituto Nacional de Estadística [INE] (2019, p. 16) survey’s methodological report states that its questionnaire is primarily based on the GGS project, enhancing cross-country comparability by ensuring consistent variable and concept alignment.

However, working with distinct data sources presents challenges, such as potential differences in survey design, question wording, and data collection methods, which affect the comparability of results. The temporal gap (2018 for Spain, 2021 for Finland/Germany) might also reflect contextual changes (economic conditions, policy environments) influencing fertility intentions. We acknowledge that broader socioeconomic conditions, such as fluctuations in the labor market or the differential early impacts of the COVID-19 pandemic (relevant for the GGS 2021 data collection period), could play a role (Guetto et al., 2022). We interpret our findings with this caution, focusing on more enduring structural patterns rather than short-term conjunctural effects. To ensure comparability, we conducted a variable harmonization process that aligned key variables conceptually and methodologically. The details of this process are provided in the Appendix 1. While acknowledging these limitations, we carefully addressed them when interpreting the findings to minimize potential biases.

From these datasets, we selected the subsample of analytical units most relevant for analyzing fertility intentions: individuals aged 18 to 49 who reported being partnered (including cohabiting and non-cohabiting couples) at the time of the survey. This age range aligns with social childbearing norms; individuals outside this age range are excluded because their intentions are likely less influenced by economic, occupational, or gender equality factors and more by age-related social norms (Badolato et al., 2024). Focusing on partnered individuals reflects the study’s emphasis on fertility intentions within a relational context (Sturm et al., 2023). The extracted subsamples^1^ comprise 2,229 cases from Finland, 16,699 cases from Germany, and 12,560 cases from Spain.

The analytic approach involves separate models for men and women, acknowledging gender-based differences in the consequences of parenthood. Both databases are of high quality, providing comprehensive data on gender relations, family dynamics, and fertility intentions, with differentiated information for men and women, making them well-suited for our research objectives.

### 3.2 Variables

The dependent variable, short-term fertility intentions, is operationalized with the question: ‘Do you intend to have a/another child during the next 3 years?’ Responses were binary: ‘yes’ (including ‘probably/definitely yes’) and ‘no’ (including ‘probably/definitely not’). ‘Unsure’ cases (12.56% in Finland, 14.83% in Germany) were excluded as they lack clear positive/negative intent, which would introduce ambiguity for CART models. In Spain, the dependent variable does not include an ‘Unsure’ category. While ensuring analytical clarity, we acknowledge that individuals with uncertain characteristics may have distinct characteristics, which is a potential area for future research using different techniques.

Drawing from the theoretical and empirical insights discussed in the preceding section (2.2), independent variables are grouped into four dimensions. This structure is not merely theoretical but also methodological: the variables have been selected and coded to maximize CART’s capacity to detect interactions and thresholds. For instance, the use of categorical variables for employment status enables the algorithm to create divisions that reflect labor market segmentation, whereas continuous scales such as index care are ideal for CART to identify precise cut-off points in caregiving equity that influence intentions. The four dimensions are: (1) employment circumstances, (2) financial situation, (3) distribution and satisfaction with domestic/caregiving responsibilities, and (4) family and gender attitudes.

The first dimension, which encompasses employment circumstances, attempts to capture the role of economic stability in fertility intentions. The inclusion of both interviewee and partner employment status and contract type is crucial for examining, for example, the prevalence and impact of dual-earner models (expected to be central in Scandinavian regimes) versus male-breadwinner models (potentially more influential in Mediterranean regimes). This dimension includes the following variables: the interviewee and their partner’s employment status (employment: employed-1 or not-0), the interviewees and their partner’s work contract type (work contract: permanent-2, temporary-1, or no written contract-0), and the interviewee’s work schedule (work time: full-time-1 or part-time-0). The inclusion of both categorical (e.g., contract type) and binary variables allows CART to test how labor market precariousness interacts with other dimensions, revealing if its effect is conditional on, for example, the partner’s income or caregiving distribution.

The second dimension, financial situation, assesses the impact of economic resources. Perceived financial difficulties and household income are crucial for understanding how economic constraints on fertility intentions might be amplified in regimes with less state support (e.g., Mediterranean) compared to those with comprehensive welfare (e.g., Scandinavian), which can buffer individual financial situations. The model is operationalized through the following variables: the interviewee’s perception of financial difficulties, measured as difficulties in making ends meet (difficulties: yes-1, no-0), and monthly net household income (net income). Net household income measured in intervals differs between GGS (annual) and INE (monthly). To enable cross-model comparability, we harmonize by calculating interval midpoints and deriving an average monthly household income.

The third dimension, distribution and satisfaction with housework and caregiving, explores gendered labor divisions. We created an indicator (index care) to measure the interviewee’s perception of their involvement in childcare tasks, derived from questions regarding the usual performer of specific childcare tasks: “dressing children,” “dropping/picking them up from school,” “caring for them when sick,” “helping with homework,” and “playing with them.” A value of 1 is assigned when the interviewee claims to usually perform the task, 0.5 when they assert that both partners share the responsibility equally, and 0 when they state that their partner assumes the responsibility. The resulting index ranges from 0 (where the partner performs all tasks) to 1 (where the interviewee performs all tasks), with 0.5 indicating an equal share. This indicator captures perceived imbalances in childcare task distribution. This 0–1 scaling is particularly powerful for CART, as it allows the algorithm to pinpoint the exact perceived participation threshold (e.g., a woman doing more than 70% of tasks) where intentions shift, thus operationalizing empirically the theoretical concept of the “second shift’s” impact.

Aditionally, we have include interviewee satisfaction with household task allocation (housework_satisfaction) and childcare responsibility distribution (childcare_satisfaction) on a 0–10 scale. Measuring actual childcare task distribution (via index care) and satisfaction with these arrangements also allows testing hypotheses about the “second shift” and its impact on fertility intentions, which may vary by normative expectations and institutional support for care equality within each regime (e.g., similar care imbalance might be perceived differently or have different consequences in Scandinavian vs. Mediterranean contexts).

The fourth dimension, family and gender attitudes, accounts for cultural and attitudinal factors. First, we have formulated an index (index values) to measure conservatism-progressivism on this front because they are expected to reflect the differing “gender contracts” and cultural underpinnings of each regime, which shape the normative context for fertility decisions (e.g., traditional values are potentially stronger in Mediterranean regimes, and progressive or secular in Scandinavian regimes). This index is derived from a set of questions gauging the interviewee’s degree of agreement or disagreement with specific scenarios: the right to divorce, the rights of same-sex couples, the necessity of marriage for cohabitation, the importance of a mother and father for a child’s happiness, and the need for men and women to have children to feel fulfilled. A value of 1 is assigned when the interviewee agrees with the right to decide or equality, and 0 when they express disagreement, with adjustments made for the direction of each item’s formulation. The resulting index ranges from 0 to 1, with higher scores indicating greater progressivism. The continuous nature of this index enables CART to identify value clusters (e.g., individuals with scores below 0.6) that, in interaction with structural factors, predict fertility intentions, testing hypotheses about how ‘gender contracts’ operate at the micro-level. Second, religiosity was included as a predictor (religion), coded as 1 for respondents who identified as religious and 0 for those who did not identify as such.

Other relevant sociodemographic variables included age, cohabitation status (cohabit), marital status (marriage), educational levels of the interviewee and their partner (education), and the number of children (children, considering both biological and adopted, as well as current and previous partners). Table 1 summarizes the variables, operationalization, descriptive statistics, and final sample sizes per country and gender after the selection process.

**Table 1:** Summary of descriptive statistics and sample sizes for variables analyzed in Finland, Germany, and Spain by sex.

Variable	Categories	Descriptives					
		Finland		Germany		Spain	
		Men	Women	Men	Women	Men	Women
Childbearing_intention *(intention to have a/another child in the next 3 years)*	Yes (1)	17.4%	20.4%	17.9%	20.1%	18.0%	17.4%
	No (0)	82.6%	79.6%	82.0%	79.9%	81.9%	82.6%
Dimension 1. Employment circumstances							
Employment *(employment status)*	Yes (1)	73.4%	69.8%	77.6%	73.6%	74.9%	63.1%
	No (0)	26.7%	30.3%	22.4%	26.4%	25.1%	36.9%
Work_time *(work time)*	Full-time (2)	65.4%	53.2%	71.0%	39.3%	69.4%	47.4%
	Part-time (1)	4.9%	34.2%	6.1%	32.1%	5.5%	15.7%
	No job (0)	29.7%	12.1%	22.9%	28.6%	25.1%	36.0%
Work_contract *(work contract type)*	Permanent (2)	60.2%	49.7%	68.4%	61.9%	52.9%	41.6%
	Temporary (1)	7.0%	14.4%	6.4%	7.9%	32.8%	14.6%
	No contract (0)	32.7%	35.9%	25.1%	30.1%	14.4%	43.8%
Partner_employment *(partner employment status)*	Yes (1)	80.4%	82.6%	77.5%	85.9%	64.6%	83.0%
	No (0)	19.6%	17.4%	22.5%	14.1%	35.4%	16.9%
Partner_work_contract *(partner work contract type)*	Permanent (2)	61.45%	72.92%	65.23%	77.40%	45.22%	62.53%
	Temporary (1)	14.80%	7.68%	6.36%	5.33%	16.34%	16.27%
	No written contract (0)	23.74%	19.40%	28.41%	17.27%	38.45%	21.20%
Dimension 2. Household and financial situation							
Net_income *(total household net income, monthly)*	Continuous (in euros)	M: 3699.1 S: 2472.1	M: 3617.3 S: 2411.8	M: 3685.3 S: 1214.2	M: 3569.4 S: 1235.3	M: 2174.5 S: 1343.9	M: 2055.3 S: 1293.8
difficulties *(difficulties to make ends meet)*	Yes (1)	18.1%	23.4%	17.6%	20.1%	41.9%	56.2%
	No (0)	81.9%	76.6%	82.4%	79.9%	58.1%	43.8%
Dimension 3. Division of housework and care work							
Housework_satisfaction *(satisfaction with housework division)*	Scale 0–10	M: 8.3 S: 1.5	M: 7.8 S: 1.9	M: 8.1 S: 1.8	M: 7.9 S: 2.4	M: 8.5 S: 1.8	M: 7.2 S: 2.6
Index_care *(perceived balanced/inbalanced division)*	Scale 0–1	M: 0.4 S: 0.2	M: 0.6 S: 0.2	M: 0.3 S: 0.2	M: 0.7 S: 0.2	M: 0.4 S: 0.2	M: 0.6 S: 0.2
Childcare_satisfaction *(satisfaction with childcare tasks division)*	Scale 0–10	M: 8.7 S: 1.2	M: 8.2 S: 1.6	M: 8.3 S: 1.6	M: 7.6 S: 2.2	M: 8.8 S: 1.5	M: 7.8 S: 2.4
Dimension 4. Gender and family values							
Religion *(believing in any religion)*	Yes (1)	57.0%	66.0%	55.9%	63.6%	63.7%	73.3%
	No (0)	42.9%	34.0%	44.1%	36.4%	36.2%	26.7%
Index_values *(conservatism-progressivism regarding family and gender index)*	Scale 0–1	M: 0.8 S: 0.2	M: 0.9 S: 0.2	M: 0.7 S: 0.2	M: 0.8 S: 0.2	M: 0.8 S: 0.2	M: 0.8 S: 0.2
Sociodemographics							
Age	Continuous (18–59)	M: 34.9 S: 8.9	M: 33.4 S: 8.5	M: 34.6 S: 8.8	M: 34.4 S: 8.8	M: 38.6 S: 10.6	M: 39.2 S: 10.6
Partner *(having a partner)*	Yes (1)	67.6%	76.3%	72.8%	79.6%	70.4%	73.6%
	No (0)	32.4%	23.7%	27.2%	20.4%	29.6%	26.4%
Cohabit *(cohabiting with partner)*	Yes (1)	58.7%	63.4%	61.1%	66.1%	58.5%	60.5%
	No (0)	41.3%	36.6%	38.9%	33.9%	41.5%	39.5%
Marriage *(married with partner)*	Yes (1)	34.4%	34.2%	60.1%	43.7%	45.7%	49.6%
	No (0)	65.7%	65.8%	39.9%	56.3%	54.3%	50.4%
Education *(educational level)*	Primary (1)	0.0%	0.1%	0.4%	0.4%	15.7%	13.1%
	Secondary (2)	47.6%	40.7%	50.3%	51.7%	42.1%	34.8%
	Tertiary (3)	52.4%	59.3%	49.3%	47.9%	42.2%	52.1%
Partner_education *(partner educational level)*	Primary (1)	0.1%	0.2%	0.4%	0.4%	12.9%	18.9%
	Secondary (2)	32.1%	50.2%	48.3%	46.7%	35.3%	38.0%
	Tertiary (3)	67.8%	49.6%	51.4%	52.9%	51.8%	43.0%
Children *(number of children)*	Continuous (0–14)	M: 0.8 S: 1.2	M: 0.9 S: 1.2	M: 0.7 S: 1.1	M: 0.9 S: 1.1	M: 0.8 S: 1.0	M: 1.0 S: 1.1

Source: Own elaboration based on data from the GGS for Finland and Germany (2021) and the INE Fertility Survey for Spain (2018), segregated by sex of the interviewee.

### 3.3 Analytical approach: decision tree models based on CART

We selected CART in this article for their ability to handle complex, non-linear variable interactions and provide interpretable decision rules, suiting the multifaceted nature of fertility intentions across diverse contexts. CART recursively splits the dataset into subsets based on feature values, creating a tree-like structure where internal nodes represent attribute decisions and leaf nodes represent class labels. The goal is to identify splits that yield the most homogeneous subgroups, thereby improving predictive accuracy. CART models are valued for their simplicity, interpretability, and ability to handle both numerical and categorical data. Incorporating variables from four key dimensions (employment, financial, domestic labor, gender, and family values) allows for a nuanced understanding of factors influencing fertility intentions. This approach ensures an accurate representation of unique attributes and interactions within each country and gender group, thereby enhancing the robustness of the findings.

We developed six separate CART models (one per country, disaggregated by sex). The output variable for our predictive models was short-term fertility intentions, coded as a binary outcome (1 = affirmative/success, 0 = negative/failure). The input variables (or predictors) were drawn from the four dimensions previously outlined: employment circumstances, financial situation, domestic/caregiving responsibilities, and family/gender values. Organizing predictors into these dimensions allows for a detailed examination of the factors influencing fertility intentions within the distinct Scandinavian, Continental, and Mediterranean gender regimes. The models were validated to assess generalizability and prevent overfitting. Given the varying sample sizes, a differentiated approach was used: 10-fold cross-validation for smaller datasets (*N* < 5,000: Finland Men *N* = 863, Finland Women *N* = 1,350, Germany Men *N* = 4,918, Spain Men *N* = 1843), and a 70/30% training/test split for larger datasets (*N* ≥ 5,000: Germany Women *N* = 6,731, Spain Women *N* = 7,505). This dual approach ensures robust, subsample-appropriate validation.

The performance of the CART model was evaluated using several metrics. Sensitivity measures the proportion of actual positives (i.e., individuals with affirmative short-term fertility intentions, coded as 1) that are correctly identified by the model. Specificity indicates the proportion of actual negatives (i.e., individuals with negative short-term fertility intentions, coded as 0) that are correctly identified. Precision tells us, of all the individuals our model predicts will have affirmative short-term fertility intentions, what proportion of those predictions are correct (meaning, those individuals do have affirmative intentions). Finally, the Area Under the Receiver Operating Characteristic Curve (AUC-ROC) summarizes the overall discrimination ability of the model across all possible classification thresholds. Together, these metrics provide a comprehensive assessment of the model’s predictive accuracy for short-term fertility intentions.

Table 2 then presents the classification performance of the CART models developed for each country. These tables, often referred to as confusion matrices, detail how accurately the models predict short-term fertility intentions (distinguishing between affirmative, ‘1’, and negative, ‘0’, intentions) compared to the actual observed intentions. The table also includes key performance metrics such as sensitivity, specificity, precision, and the AUC-ROC, which collectively assess the predictive power and discriminatory ability of each model. In the following sections, we will delve into the specific factors identified by these models as being most strongly associated with childbearing intentions.

**Table 2:** Summary of the “short-term fertility intentions” dependent variable distribution in the models for Finland, Germany, and Spain.

	Finland		Germany				Spain		
	Men	Women	Men		Women		Men	Women	
			Training	Test	Training	Test		Training	Test
Yes (1)	200 (23.2%)	345 (25.6%)	1,081 (21.9%)	464 (21.9%)	1,536 (22.8%)	647 (22.4%)	361 (19.6%)	1,367 (18.2%)	629 (19.6%)
No (0)	663 (76.8%)	1,005 (74.4%)	3,837 (78.1%)	1,654 (78.1%)	5,195 (77.2%)	2,244 (77.6%)	1,482 (80.4%)	6,138 (81.7%)	2,583 (80.4%)
All	863 (100%)	1,350 (100%)	4,918 (100%)	2,118 (100%)	6,731 (100%)	2,891 (100%)	1843 (100%)	7,505 (100%)	3,212 (100%)

Source: Own elaboration based on data from the GGS for Finland and Germany (2021) and the INE Fertility Survey for Spain (2018), segregated by sex of the interviewee.

## 4 Results

This section examines the primary determinants of short-term fertility intentions in Finland, Germany, and Spain, as identified by CART models. To capture the influences of gender and national context, analysis is conducted separately for men and women in each country. Results first examine decision tree structures, emphasizing the most influential variables and their interactions, thereby uncovering distinct patterns characterizing fertility intentions across different gender regimes. The section concludes by assessing the models’ predictive performance.

### 4.1 CART decision trees and relative variable importance analysis

**Figure 2:**
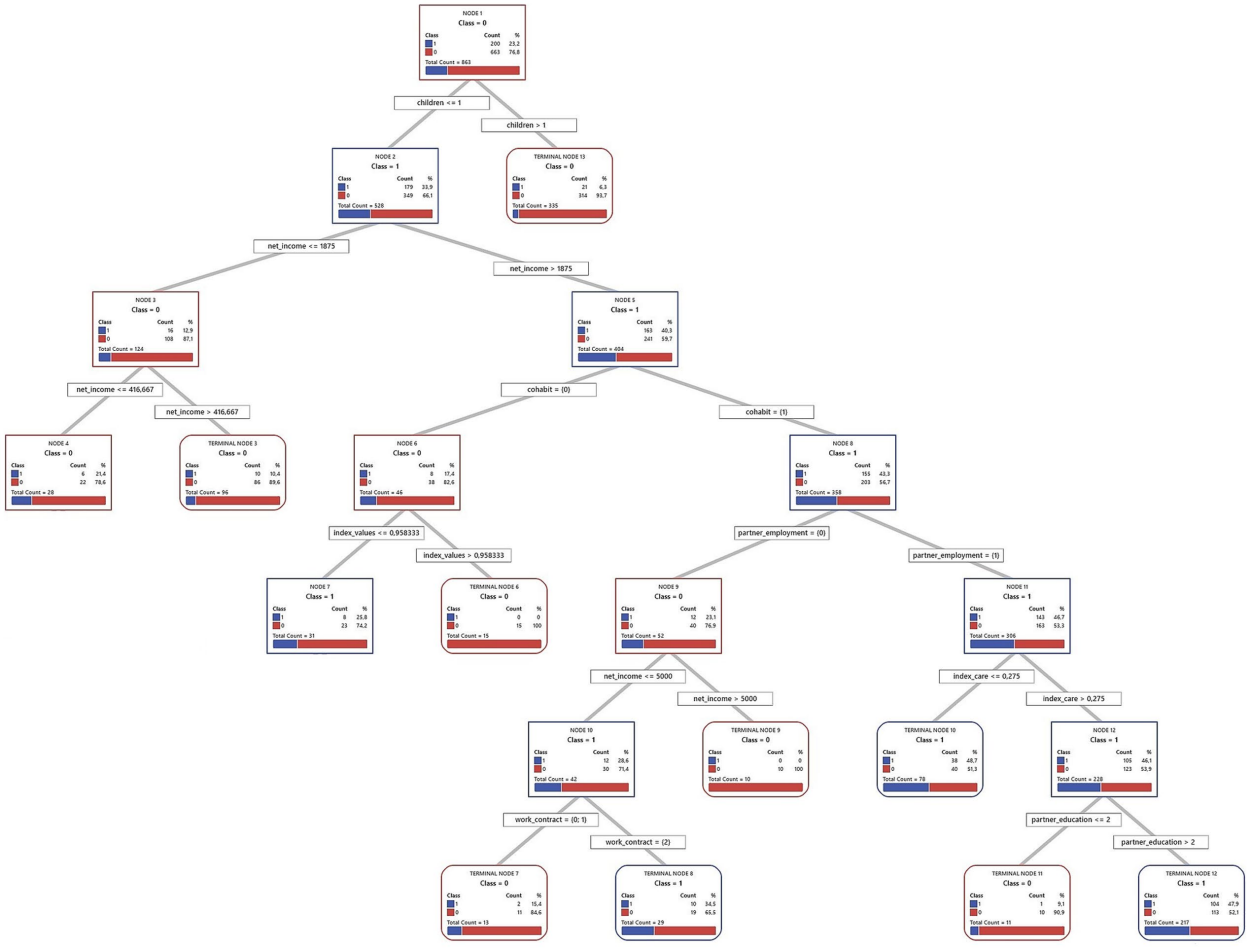
Decision tree diagram for short-term fertility intentions among men in Finland using CART models (*N* = 863). Source: Own elaboration based on data from the GGS for Finland (2022), segregated by sex (men).

**Figure 3:**
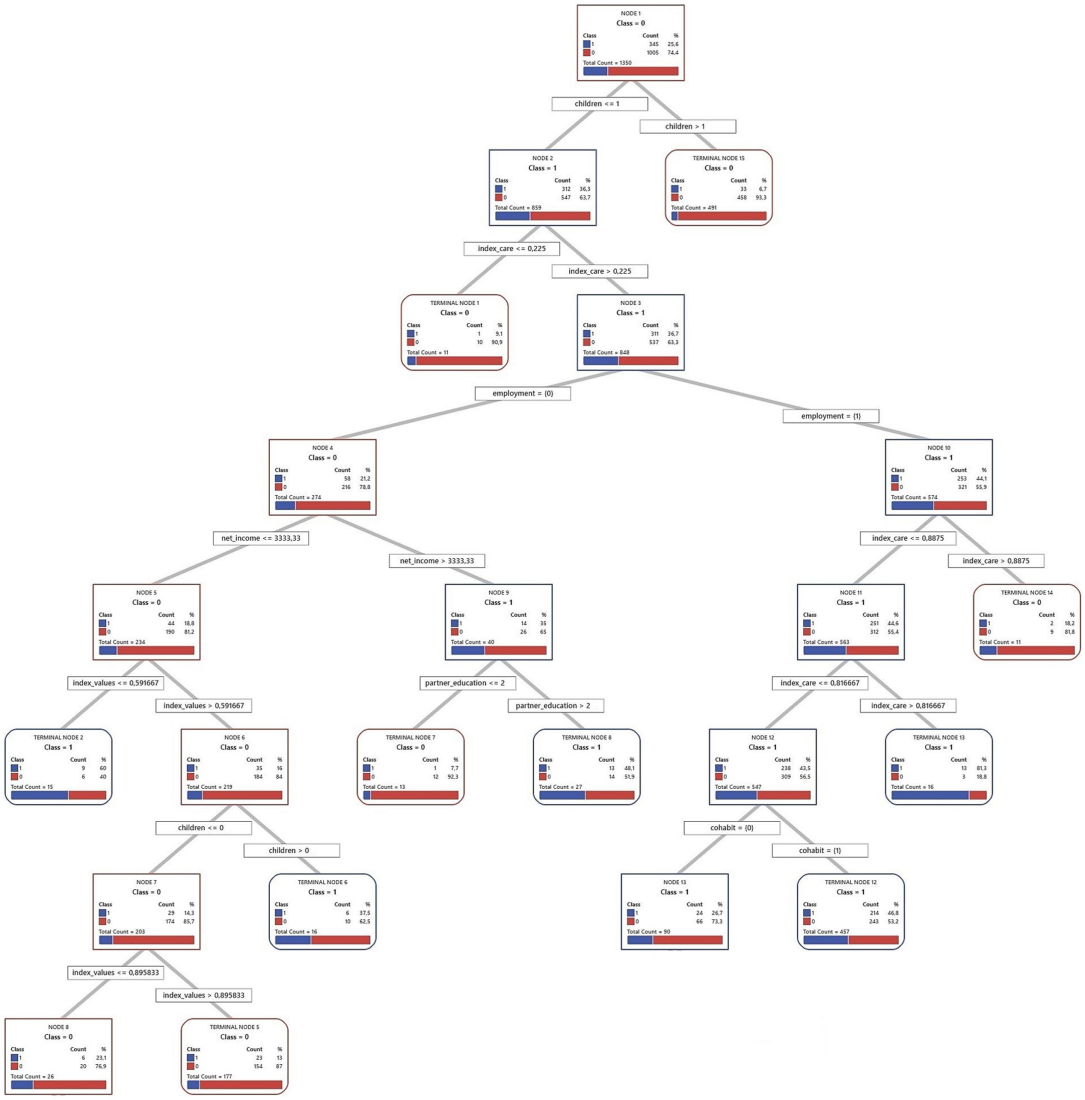
Decision tree diagram for short-term fertility intentions among women in Finland using CART models (*N* = 1,350). Source: Own elaboration based on data from the GGS for Finland (2022), segregated by sex (women).

**Figure 4:**
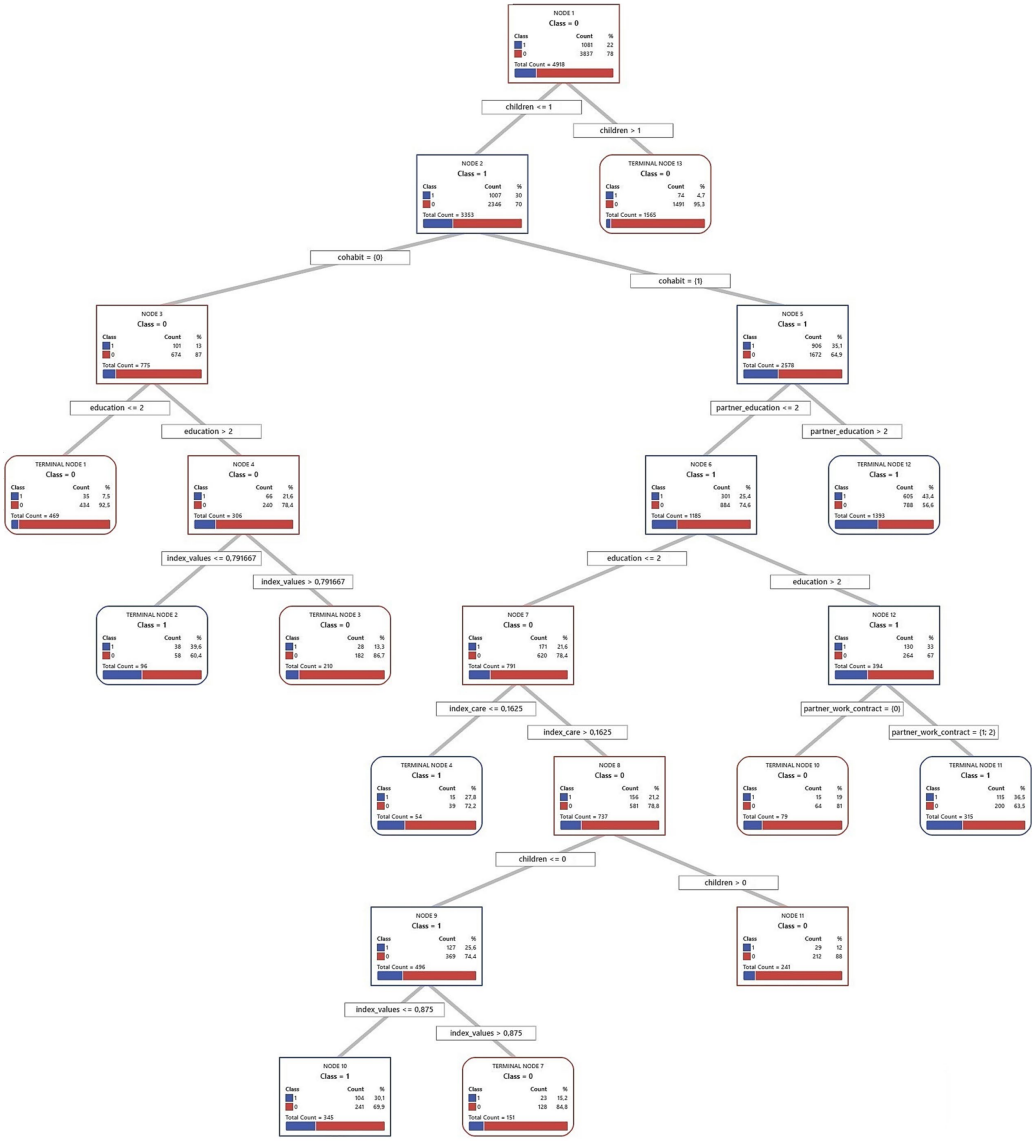
Decision tree diagram for short-term fertility intentions among men in Germany using CART models (*N* = 4,918). Source: Own elaboration based on data from the GGS for Germany (2022), segregated by sex (men).

**Figure 5:**
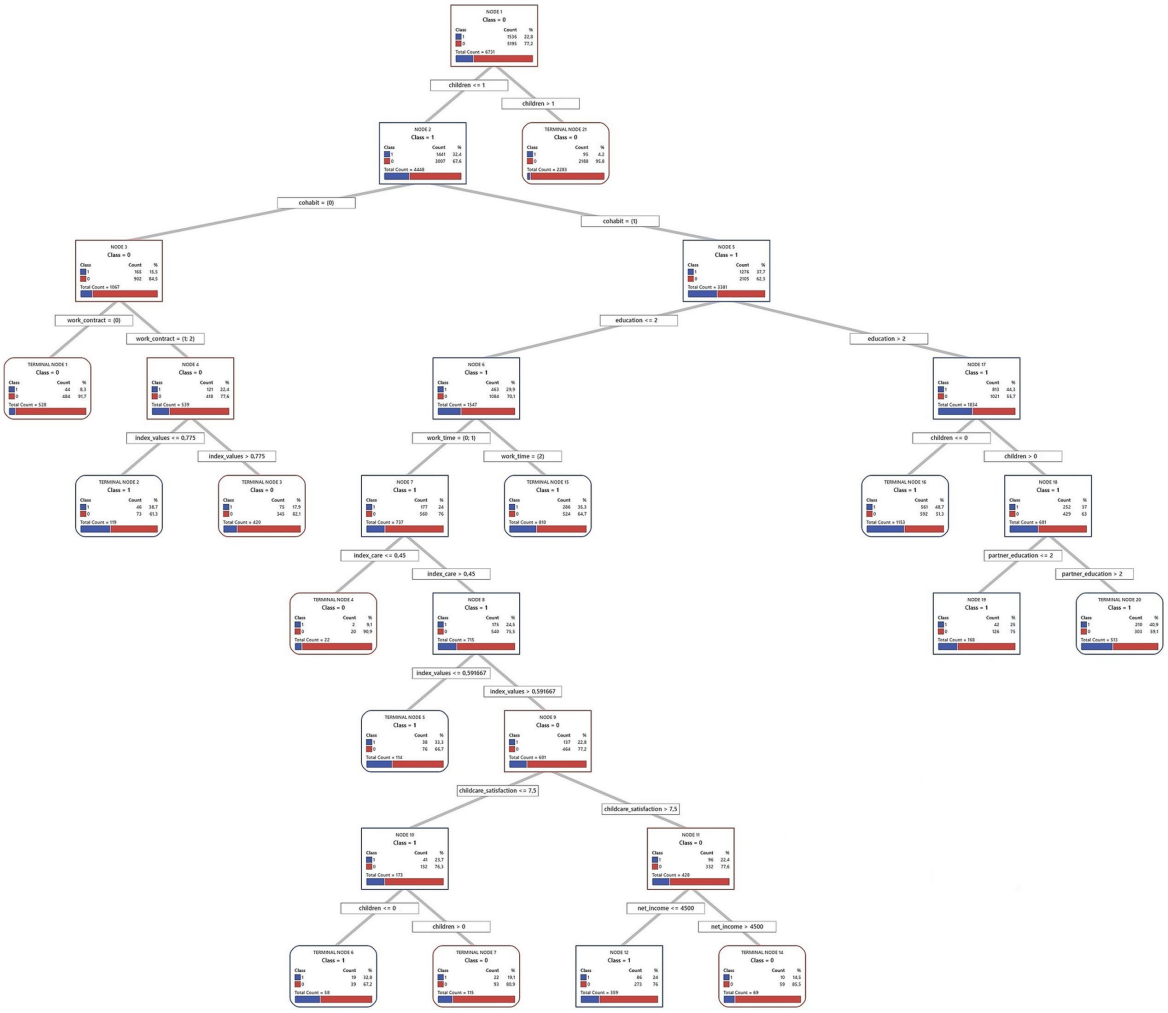
Decision tree diagram for short-term fertility intentions among women in Germany using CART models (*N* = 6,731). Source: Own elaboration based on data from the GGS for Germany (2022), segregated by sex (women).

**Figure 6:**
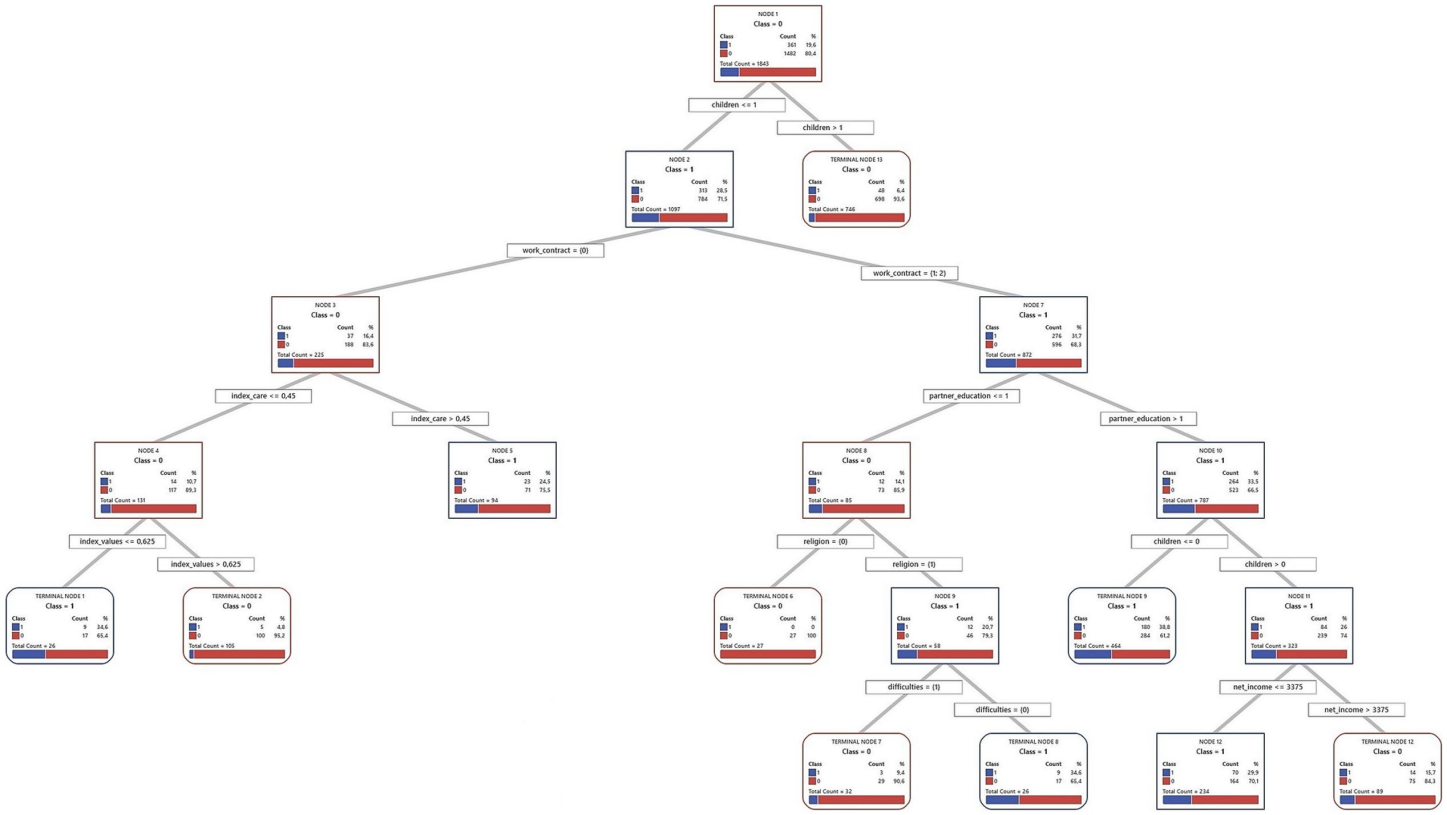
Decision tree diagram for short-term fertility intentions among men in Spain using CART models (*N* = 1843). Source: Own elaboration based on data from the INE fertility survey for Spain (2018), segregated by sex (men).

**Figure 7:**
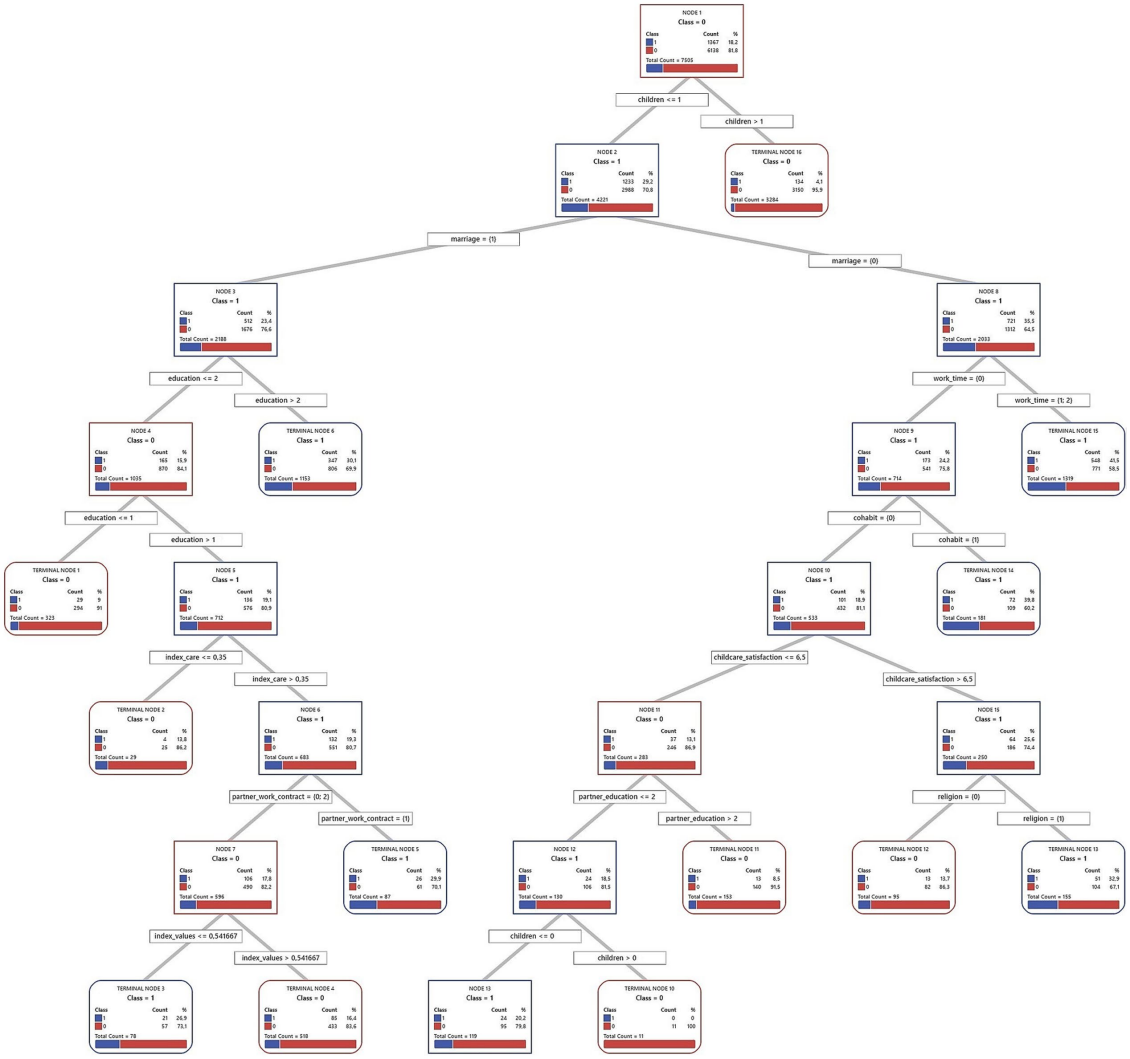
Decision tree diagram for short-term fertility intentions among women in Spain using CART models (*N* = 7,505). Source: Own elaboration based on data from the INE fertility survey for Spain (2018), segregated by sex (women).

Decision trees from the CART algorithm were used to identify and rank key factors predicting short-term fertility intentions. These models iteratively divide the sample into increasingly homogeneous subsets based on the predictor variables, creating a hierarchical structure, which is presented in full in Figures 2–7 for each country and gender. Given the complexity of these trees, the following textual analysis will focus on highlighting the most influential variables, key decision pathways, and the profiles associated with the highest and lowest probabilities of positive fertility intentions rather than providing an exhaustive description of every node and split.

**Figure 8:**
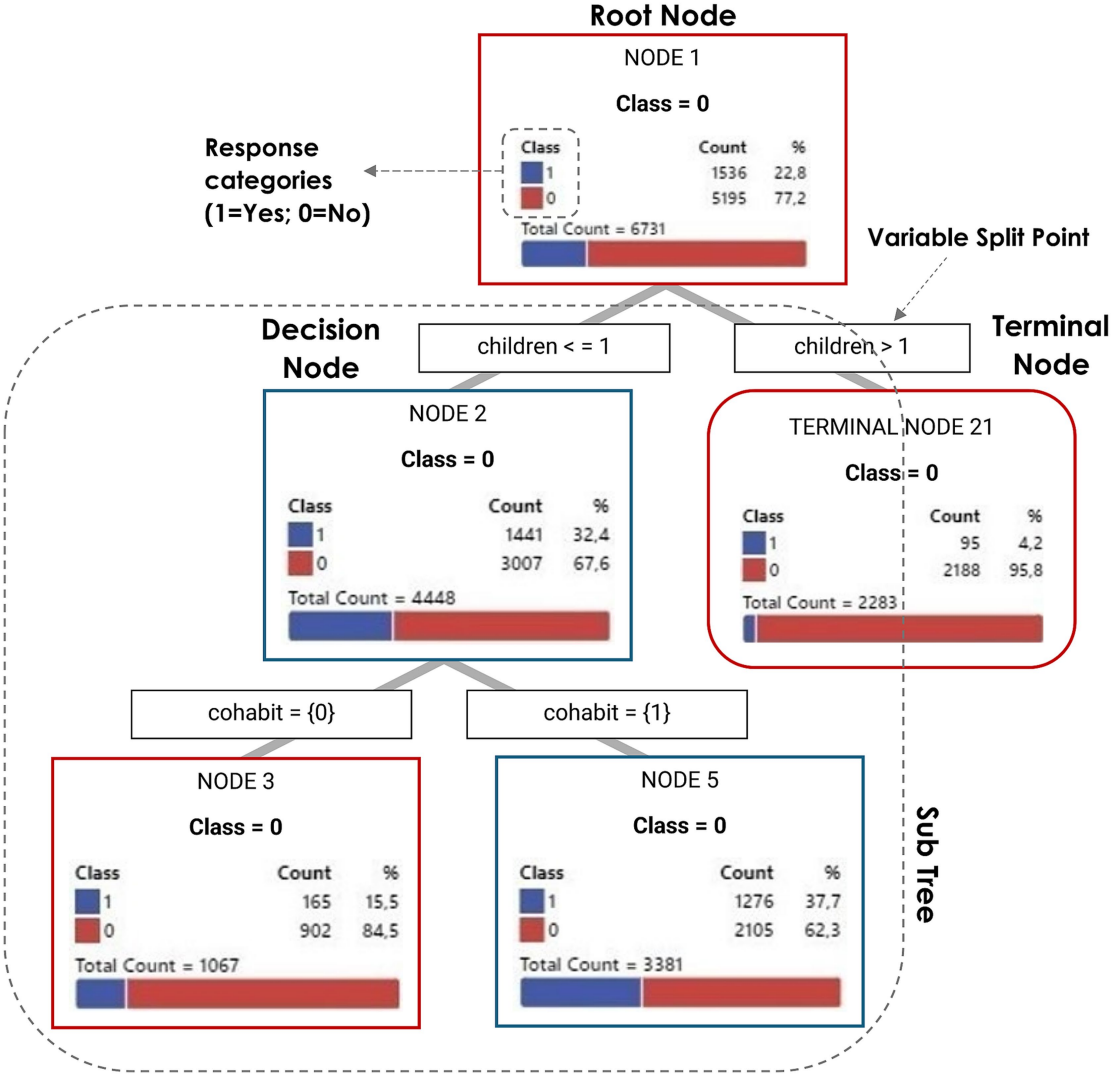
Structure of a decision tree illustrating key components. Source: own elaboration.

As discussed theoretically, factors like caregiving, employment, and gender values are central to understanding the fertility ideal-intention gap. CART models empirically test these theoretical assumptions by showing how these variables interact and influence intentions within different gender regimes. Figure 8 illustrates a decision tree’s main components (root, decision, and terminal nodes; variable split points; response categories), including a subtree example and class distribution per node.

Each node represents a decision based on a specific variable’s threshold, with branches indicating sample divisions based on that variable’s values. Nodes split until terminal nodes (“leaves”) are reached, indicating sufficient homogeneity or a subset too small for further splitting. The terminal nodes provide the final classifications, reflecting the probability of positive fertility intentions within 3 years (yes/no). Predominantly blue nodes indicate a high proportion of positive short-term fertility intention (event level), while red nodes indicate a high proportion of negative intention (non-event level). The interpretation focuses on how the predictor variables interact to influence the outcome.

The root node at the tree’s top represents the most important variable for the initial sample split. Moving down, additional relevant variables refine the classification into more specific subsets. Node percentages indicate case proportions per category, clarifying how individual characteristics affect fertility intentions.

**Figure 9:**
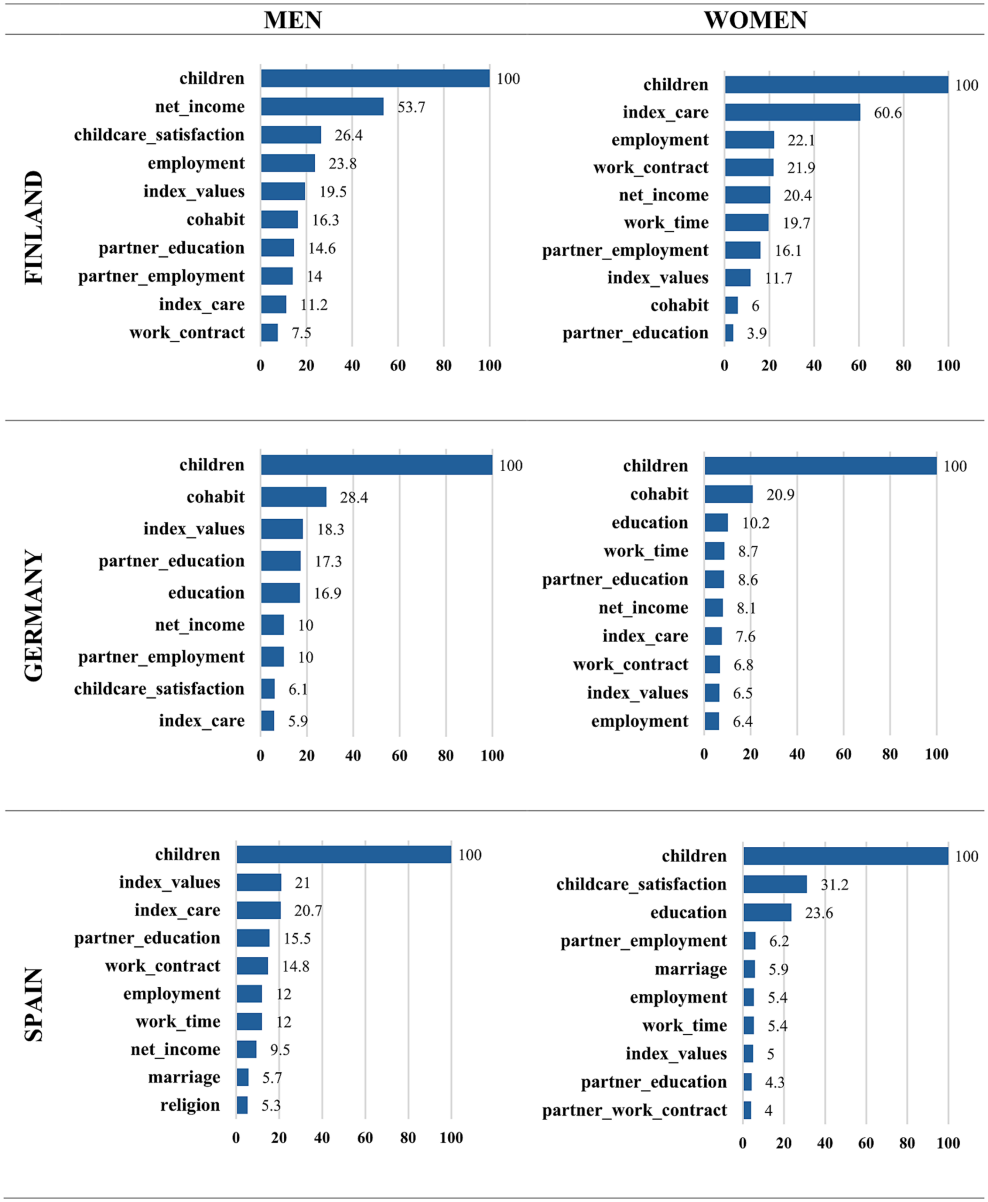
Relative variable importance included in the CART Models for Finland, Germany, and Spain by sex (%). Source: own elaboration based on data from the GGS for Finland and Germany (2021) and the INE Fertility Survey for Spain (2018), segregated by sex of the interviewee.

A variable’s relative importance is reflected by its tree position and decision node frequency. As shown in Figure 9, we analyzed the relative variable importance, which quantifies the contribution of each predictor to model accuracy. This percentage measures model improvement from the split on the predictor. Significant relative importance means that a variable is frequently used for splits, strongly influencing decision boundaries and classification, thereby enhancing the model’s ability to distinguish classes and reduce misclassification.

Across all three countries, and for both men and women, the number of existing children consistently emerged as the most powerful initial differentiating factor in the decision trees. Intentions to have another child dropped sharply for those who already had two or more children. Consequently, the following detailed discussion of the tree structures for each country (Figures 2–7) will focus primarily on the pathways observed for individuals with one child or no children, as this group exhibits more variation and complex interactions with other predictive factors.

Results for each country are structured around the same four key dimensions of independent variables previously identified: employment circumstances, financial situation, domestic and caregiving responsibilities (distribution and satisfaction), and family and gender attitudes. For each model, we considered the depth at which variables from each dimension appear and their interactions. This approach identifies profiles with varying propensities for short-term fertility intentions and determines the most influential variables for each model.

#### 4.1.1 Finland

In Finland, a country often cited as an exemplar of the Scandinavian gender regime due to its emphasis on gender equality policies and comprehensive welfare, the factors associated with short-term fertility intentions among individuals with one or no children present a complex picture. This picture may reflect interactions between the regime’s egalitarian ideals and the varied experiences at the household level, potentially related to what can be conceptualized as “gender contracts.” The full decision trees are presented in Figure 2 (for men) and Figure 3 (for women).

For Finnish men with one or no children, 33.9% express positive short-term fertility intentions. The decision tree analysis shows that the household’s financial situation is the initial key differentiator. The model identifies a threshold at €1,875 in monthly net household income, which is notably lower than the sample’s average for Finnish men (€3,699.1). This threshold effectively distinguishes those with particularly low incomes.

Men whose household income exceeds €1,875 are much more likely to intend to have a child (40.3%) compared to those below this threshold (12.9%). Within the higher-income group, cohabitation with a partner further increases the likelihood of positive intentions: 43.3% of cohabiting men express such intentions, compared to only 17.4% of non-cohabiting men.

Among cohabiting, higher-income men, the partner’s employment status becomes another important factor. If the partner is also employed, the intention rate rises to 46.7%. In contrast, if the partner is not employed, the rate drops to 23.1%. This pattern is consistent with the dual-earner family model that is prevalent in Scandinavian countries.

An important detail emerges when examining men’s perceived involvement in childcare tasks (measured by index care) within the specific subgroup of cohabiting, higher-income men whose partners are employed. The model identifies a threshold at 27.5% of perceived care involvement. This threshold means that the man perceives he undertakes slightly more than a quarter of the total five listed childcare tasks (such as dressing children, school drop-offs/pick-ups, etc.), indicating a that the partner is still perceived as the primary caregiver for most of these tasks. Men in this subgroup who perceive their contribution to these tasks as 27.5% or less show a positive intention rate of 48.7%. Conversely, those who perceive an involvement greater than 27.5% enter a branch that is further subdivided by the partner’s educational level. If the partner has a primary or secondary educational level, positive intentions drop sharply to 9.1%, whereas if the partner has a tertiary educational level, positive intentions are at 47.9%.

Notably, within this specific pathway of the tree, the highest intention rate (48.7%) is observed among men reporting lower care involvement. This is slightly higher than the 47.9% observed in men with high care involvement whose partners also have a high educational level. This dynamic suggests that while greater male care participation is often positively associated with fertility intentions in broader contexts or in combination with other factors, in this particular subgroup of Finnish men with certain socioeconomic characteristics, a perceived lower involvement in care does not diminish, and may even be associated with slightly higher intentions, compared to those who are more involved but whose partner also has a high educational level. These findings point to the complexity of negotiations and perceptions regarding the division of household labor and its link to fertility decisions.

Turning to Finnish women with one or no children, 36.3% express positive short-term fertility intentions. A crucial initial factor differentiating their intentions is their perceived share of childcare responsibilities. This perception may reflect how domestic labor is organized within their households. In this sample, Finnish women perceive, on average, that they undertake 60% of care tasks (Table 1). This already indicates that, on average, they shoulder most of these responsibilities. Indeed, the vast majority report their contribution exceeds a minimal threshold of 22%. This 22% signifies the woman perceives she performs slightly more than a fifth of the listed childcare tasks.

Among women who already perceive they manage a significant share of care (more than 22%), their employment status becomes the next key factor. This may highlight the interplay between public sphere participation and private sphere responsibilities. The group with the highest probability of positive intentions (60.0%) in the entire female sample has specific characteristics. These are women who are unemployed, have lower household incomes (≤ €3,333.33 monthly), and, critically, also hold more conservative family and gender attitudes (index values ≤ 0.59). These values indicate less agreement with progressive, egalitarian statements. This result suggests that for a subgroup of women experiencing economic vulnerability, a more traditional value orientation could be strongly associated with childbearing intentions. If explored further, this finding might offer insights into diverse motivations within an egalitarian regime. It could indicate that macro-level egalitarian policies may not uniformly translate into identical micro-level value orientations or fertility decision-making processes.

Furthermore, let us consider employed women who perceive a considerable care involvement. Specifically, those who feel they undertake between 22 and 81% of these care tasks (0.22 < index care ≤ 0.81). This range is broad: it includes perceiving an equal share (50%), approaches the female average involvement (60%), and extends to shouldering a large portion of the tasks. For these women, cohabitation is a key differentiator. If cohabiting, 46.8% have positive fertility intentions, compared to 26.7% of non-cohabiting women. This suggests an association between stable partnerships and higher intentions among working mothers managing considerable care duties, possibly reflecting a perceived need for support.

In synthesis, the Finnish models identify patterns that, in some instances, seem to align with core tenets of the Scandinavian regime, such as the association between dual-earner characteristics and men’s intentions and the salience of care distribution in women’s fertility decisions. However, they also unveil significant internal heterogeneity. The nuanced associations for male care involvement and, more prominently, the link between conservative values and high fertility intentions for a subgroup of economically vulnerable women suggest that individual circumstances and diverse value orientations are associated with fertility intentions.

#### 4.1.2 Germany

In Germany, often characterized by a conservative-corporatist gender regime with a historical emphasis on the male breadwinner model alongside more recent policy shifts toward supporting dual-earner families, the factors associated with short-term fertility intentions among individuals with one or no children reveal distinct patterns. These patterns may offer insights into the interplay between traditional societal influences and evolving individual or couple-level strategies, potentially reflecting how broader regime characteristics interact with household decisions. The full decision trees for Germany are presented in Figure 4 (men) and Figure 5 (women).

For German men with one or no children, cohabitation status is a fundamental predictor. Cohabiting men are much more likely to express intentions to have a child (35.1%) than non-cohabiting men (13.0%). Within the group of cohabiting men, those whose partners have tertiary education show an even higher intention to have a child (43.4%). This may reflect a shift from the traditional male breadwinner model toward valuing dual educational and economic contributions within the couple. Even if the partner does not have tertiary education, intentions remain relatively high (36.5%) when the man himself has tertiary education and his partner has an employment contract.

For non-cohabiting men, education and personal values play a more prominent role. Among those with tertiary education, men with relatively conservative to moderately progressive values are more likely to want a child (36.6%) than those with more progressive^2^ values (13.3%). In contrast, personal values seem less relevant for non-cohabiting men with medium or lower education, whose fertility intentions are very low (7.5%). This may indicate an accumulation of perceived disadvantages for this group.

For German women with one or no children, cohabitation is a key factor. Women living with a partner show higher short-term fertility intentions (31.7%) than non-cohabiting women (15.5%). If a cohabiting woman has tertiary education, her desire for children increases, with 44.2% expressing positive intentions. Indeed, the highest fertility intentions in this entire model (48.7%) are found among cohabiting women with tertiary education and no children. If these tertiary-educated women already have one child (registering 37% intentions), their partner’s education also matters. Those whose partners also have tertiary education show higher intentions (40.9%) than those with less-educated partners (25%). This suggests a “dual-status couple” model, where both partners’ high educational attainment boosts fertility intentions (Walper and Kreyenfeld, 2022). This shared educational status may improve earnings, facilitate childrearing planning, or align family goals.

Now, let us consider cohabiting German women with medium or lower education (with 29.9% intentions in this initial group) and one or no child. Here, employment status and perceived care distribution reveal interesting dynamics. It is relevant to note that, on average, German women in this sample perceive they undertake 70% of care tasks, indicating a high overall involvement. Those working full-time have higher fertility intentions (35.3%). For women not working full-time (in part-time work or unemployed), who collectively show 24% intentions, care distribution is crucial. Most of them (715 out of 737 cases in this situation) feel care is fairly balanced or that they shoulder most of it (undertaking more than 45% of tasks), and this group presents a 22.8% rate of positive intentions. This 45% threshold, while implying a significant contribution from the woman, is below the female average involvement (70%) and suggests a perception of a care division closer to equality compared to the general norm of high female involvement. In contrast, a small group (22 cases) feeling a greater imbalance (undertaking 45% or less) has much lower intentions (9.1%).

For the majority group of women not in full-time employment but perceiving care involvement above 45%, their gender and family values (index values) are the next important factor. The cut-off is 0.59. Those with more conservative values show notably higher fertility intentions (33.3%) than those with more egalitarian values, who present an intention rate of 22.8%. This suggests that for German women with medium/lower education, not working full-time but with a perceived fair care division (or at least not as imbalanced as the general average), a more traditional outlook is linked to a stronger desire for more children. German policies supporting maternal part-time work may interact with couple-level agreements on care and individual attitudes, thus affecting fertility intentions.

In summary, the exploratory analysis for Germany suggests that fertility decisions are navigated within a framework where stable unions and educational capital for both partners appear highly salient. However, the gendered division of labor, particularly concerning care, emerged as a crucial and often challenging aspect, especially for women. These observations may reflect ongoing negotiations between traditional expectations and aspirations for gender equality (Miller et al., 2021), characteristic of a transforming Continental regime. The findings suggest that while educational attainment is increasingly important for both genders, the realization of fertility intentions for women, particularly those not in full-time employment, is associated with the perceived fairness of care distribution within the partnership.

#### 4.1.3 Spain

In Spain, often characterized as a Mediterranean gender regime facing significant societal shifts, the patterns may offer insights into the complex interplay between traditional influences, emerging family formation strategies, and the persistent challenges of a “stalled gender revolution.” The full decision trees for Spain are presented in Figure 6 (men) and Figure 7 (women).

The analysis of fertility intentions among Spanish men with one or no children (Figure 6) reveals complex dynamics, possibly reflecting a gender regime in transition. Initially, employment status acts as a primary filter: employed men show higher short-term fertility intentions (31.7%) than unemployed men (16.4%). This initial distinction might align with more traditional gender roles, where male employment stability is perceived as a prerequisite for family formation. However, subsequent subdivisions nuance this view. The highest fertility intentions (38.8%) are observed in employed; childless men whose partners have medium or higher education. This finding suggests an increasing valuation of the partner’s educational capital. Such a trait could indicate a shift toward dual-earner family models or greater symmetry in spousal resources, characteristic of societies in transition.

For employed men whose partner has primary education, religion emerges as a relevant factor. Men identifying as religious show a 20.7% fertility intention, compared to 0% for non-religious men. Although this finding is based on a small subsample (85 cases), it might point to the persistence of traditional influences within specific population niches, coexisting with more modernizing trends. When employed men already have a child, household income introduces another analytical layer. The average net income for men in Spain is €2174.5. The model sets a cut-off point above this average. It is analytically interesting that men with incomes up to this (already high) threshold show a higher probability of wanting another child (29.9%) than those with even higher incomes (15.7%). This pattern challenges simplistic economic explanations. It could reflect how, in a changing context, family aspirations do not scale linearly with income beyond a certain level of well-being, perhaps due to the emergence of other attitudes or priorities.

Finally, for the group of unemployed men, with an overall fertility intention of 16.4%, their perceived involvement in childcare (index care) is important. The average perception of this for Spanish men is 40%. The model uses a 45% cut-off, where those who perceive an involvement greater than 45% (above the male average) show a 24.5% probability of positive intentions. This might indicate that a greater perceived domestic involvement, even without the traditional provider role, can sustain paternity desires. In contrast, for unemployed men who perceive care involvement of 45% or less, their personal gender attitudes (index values) become determinant. The average index values for Spanish men is 0.8 (higher values are more egalitarian). The model splits them at 0.62. It is observed that men with more conservative attitudes (index values ≤ 0.62, more traditional than average) exhibit a notable 34.6% fertility intention, while those with more egalitarian attitudes (index values > 0.62) have very low intentions (4.8%). In the absence of employment, and with a low perceived care involvement, an adherence to more traditional gender attitudes appear associated with a stronger desire for children. This could be interpreted to reaffirm traditional family roles when other markers of masculinity (like employment) are absent.

Turning to the findings for Spanish women with one child or none, these may offer further indications of potential shifts in family formation patterns. While marriage remains relevant in the Spanish context, a striking general pattern points toward higher positive short-term fertility intention among non-married women (35.5%) than among married ones (23.4). Among non-married women, economic independence and partner relationship stability are key. Employed non-married women exhibit the highest fertility intention rate in the model (41.5%), while non-employed women who are cohabiting also show high intentions (39.8%). These findings point to the consolidation of alternative pathways to motherhood outside of marriage. For other non-married women (neither employed nor cohabiting, with an 18.9% intention rate), factors such as childcare satisfaction and religiosity can play a role, with religious women reporting high childcare satisfaction showing a 32.9% intention rate.

In contrast, for married women, education is an important factor: those with tertiary education show a 30.1% intention rate, whereas for those with only primary education, it is just 9.0%. The situation for married women with secondary education is more complex and illustrates the tensions of a “stalled gender revolution.” Their fertility intentions depend on their perceived distribution of care (index care), their partner’s employment situation, and their own gender values (index values). If they perceive they perform 35% or less of care tasks (index care ≤ 0.35), their intention rate is low (13.8%). If they perceive they perform more than a third of care tasks (index care > 0.35) and their partner has a temporary contract, the intention rate rises to 29.9%. However, if, with this same perception of performing more than a third of care tasks, their partner is unemployed or has a permanent contract, intentions vary: it is 26.9% for women with more conservative gender values (index values ≤ 0.54) and 16.4% for those with more egalitarian values (index values > 0.54). This latter finding suggests that more egalitarian women might be more reluctant to have children in contexts of partner employment uncertainty if co-responsibility is not ensured.

In summary, the model for Spanish women shows how economic autonomy and cohabitation open new pathways to motherhood outside marriage. However, within marriage, especially for women with intermediate education levels, the persistence of care inequalities and their interaction with partner employment stability and women’s gender values remain determinant.

The exploratory analysis for Spain highlights specific empirical patterns that contribute to the idea that Spain might be viewed as a Mediterranean model undergoing transition: for instance, the relatively high intentions of employed non-married women, the apparent viability of cohabitation for non-employed women, and the nuanced role of a partner’s education for men. The pathways to positive fertility intentions visualized in Figures 6, 7 appear more diversified than a “classic” Mediterranean script might predict. Traditionally, such a script would emphasize marriage as the primary context for childbearing, a strong male breadwinner model, and fertility decisions heavily influenced by the husband’s economic stability and more traditional family values, with less emphasis on female employment or cohabitation as viable routes to parenthood. Our findings, however, suggest a more varied landscape. This observed transition is occurring within a challenging landscape characterized by precarious employment, a high incidence of involuntary part-time work for women, and lingering cultural expectations around female primary caregiving. These unresolved tensions, facets of an incomplete journey toward gender equality, are subtly reflected in the conditional and sometimes complex pathways to positive fertility intentions identified in this analysis.

### 4.2 Model performance and predictive accuracy

The performance of the CART models was evaluated using the following key metrics: sensitivity, specificity, precision, and the Area Under the Receiver Operating Characteristic Curve (AUC-ROC). To ensure clarity for readers unfamiliar with these terms, we provide a brief explanation: sensitivity refers to the model’s ability to identify individuals with positive fertility intentions correctly; specificity indicates the model’s ability to identify those without such intentions correctly; precision reflects the accuracy of the model’s positive predictions; and the AUC-ROC provides an overall measure of the model’s ability to discriminate between individuals with and without positive fertility intentions. Generally, AUC-ROC values above 70% are considered to indicate acceptable predictive performance.

**Figure 10:**
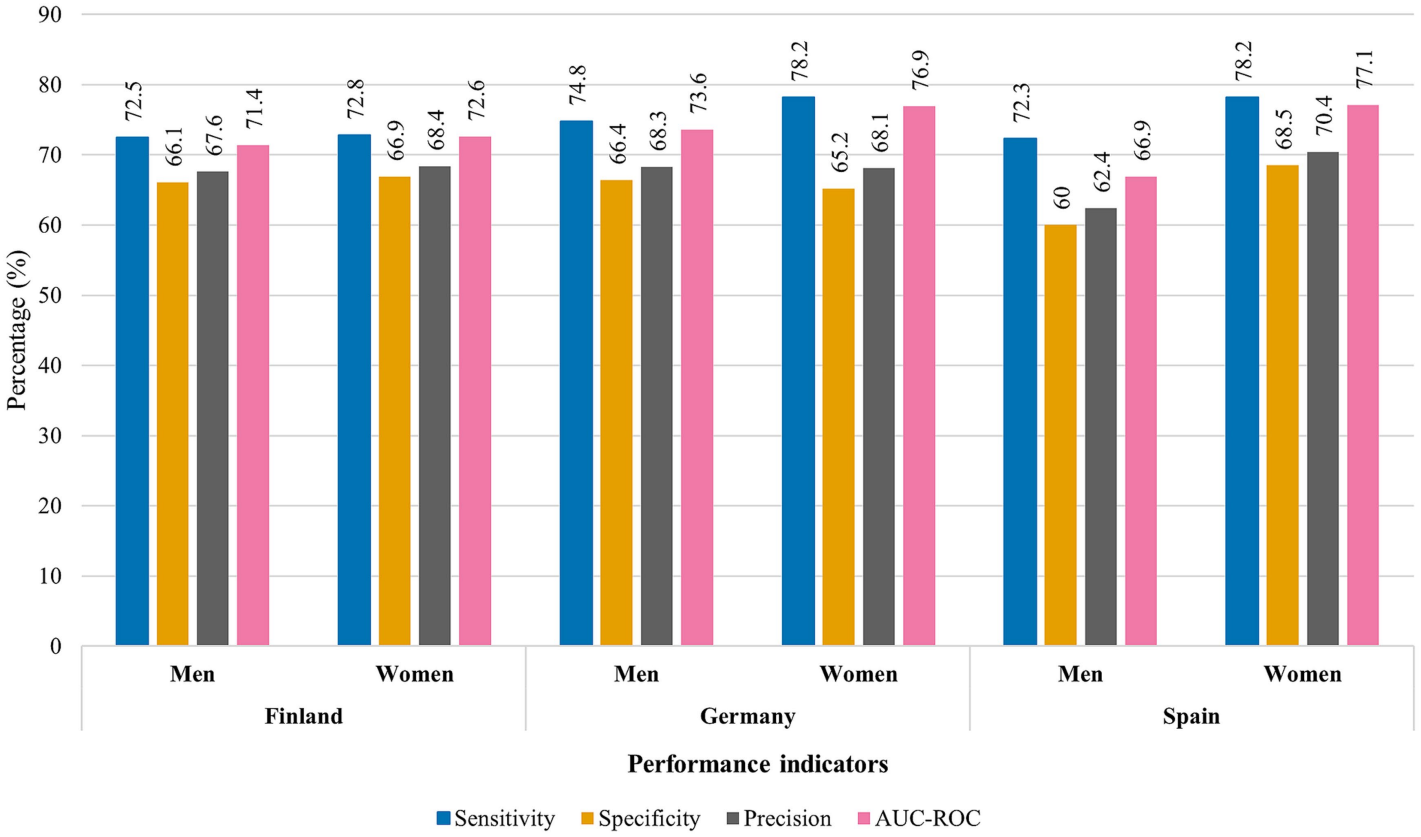
Performance evaluation of CART models for Finland, Germany, and Spain by sex. Source: Own elaboration based on data from the GGS for Finland and Germany (2021) and the INE fertility survey for Spain (2018), segregated by sex of the interviewee.

As shown in Figure 10, the models demonstrated acceptable overall predictive performance. The AUC-ROC values ranged from 71.4 to 77.1% across all groups, indicating a reasonable level of accuracy in predicting short-term fertility intentions. The models tended to be more accurate in predicting fertility intentions for women than for men. For example, sensitivity, which measures the proportion of correctly identified positive fertility intentions, was consistently higher for women across all countries (ranging from 72.9 to 78.2%) compared to men (ranging from 72.5 to 74.8%). These values suggest that the model may more readily capture the factors influencing women’s fertility intentions than those affecting men’s intentions.

While the models demonstrate acceptable predictive capabilities, it is important to acknowledge the limitations arising from the imbalanced distribution of short-term fertility intentions, where negative cases significantly outnumber positive cases. This imbalance can potentially introduce bias, leading the model to overemphasize patterns representative of the majority class and potentially limiting its ability to generalize to new data. However, the primary objective of this study was not to achieve maximum predictive accuracy but rather to gain insights into the complex interplay of factors and their relative importance in shaping fertility intentions. The CART methodology’s ability to handle non-linear relationships and visualize decision-making processes makes it a valuable tool for understanding these dynamics, even with the limitations posed by the data distribution.

## 5 Discussion

This discussion addresses our core research questions on the individual factors shaping short-term fertility intentions and how these determinants differ between men and women across distinct gender regimes. By comparing Finland, Germany, and Spain, we aim to illuminate the social, economic, and cultural processes influencing reproductive decisions in contemporary Europe. We interpret our findings not as direct causal evidence but as suggestive hypotheses about underlying mechanisms rooted in the fulfillment or frustration of gender-based needs. Crucially, we argue that these individual decisions represent a form of grassroots mobilization that collectively signals pressure for social and institutional change.

### 5.1 Key findings: short-term fertility intentions from a gender regimes comparative perspective

Our analysis, structured around the dimensions of employment, finance, caregiving, and gender/family values, reveals significant variations across Scandinavian, Continental, and Mediterranean regimes. We will highlight how financial security and employment conditions act as foundational elements, discuss the persistent impact of caregiving distribution—particularly for women in less egalitarian regimes—and explore the interplay of evolving and traditional gender values.

#### 5.1.1 The employment nexus and its influence on fertility

The relationship between employment and fertility intentions is shaped by each country’s gender regime. In Finland, findings align with the dual-earner/dual-carer model. For men in stable, higher-income unions, a partner’s employment substantially increases fertility intentions, indicating the system normalizes two incomes for family formation (Salmi and Lammi-Taskula, 2014). For employed Finnish women, cohabiting with a partner creates a conducive scenario for childbearing. However, a notable exception exists: the highest fertility intentions are found in a subgroup of unemployed, lower-income women with conservative values, suggesting alternative pathways to family formation even within a highly egalitarian regime.

Spain initially reflects the male breadwinner model, where men’s employment is a primary determinant of their fertility intentions. Yet, the data for Spanish women deviates significantly from Mediterranean expectations. The finding that non-married, employed women exhibit the highest fertility intentions challenges the assumption that female employment is secondary or detrimental to motherhood (Cukrowska-Torzewska and Matysiak, 2020), suggesting women’s economic independence is a strong facilitator of fertility aspirations. This trend reflects an “adaptive rationality” (Friedman, 2015), where women mobilize their economic independence to meet fertility goals, renegotiating the traditional gender contract.

Germany presents a hybrid “dual-status” model, where the couple’s combined socioeconomic standing is most conducive to fertility intentions. For German men, a partner’s high educational attainment is key– evidenced by the partner’s tertiary education or the man’s education with the partner’s employment contract. For German women not in full-time employment, higher intentions are critically mediated by the perceived fairness of care distribution. This is consistent with concepts like “egalitarian essentialism” (Lappegård et al., 2021) or a “modernized choice model” (Leitner, 2014), where policies may reinforce women’s primary caregiver role, making fairness in the domestic sphere a bargaining tool for decisions about further fertility.

#### 5.1.2 Financial dimension: constraints, thresholds, and relational capital

The financial dimension operates differently across countries. In Spain, variables that reflect immediate economic hardship or income levels are especially important, particularly for women. The decision tree analysis shows that lower net household income or perceived difficulties in making ends meet are linked to significantly lower fertility intentions. This finding suggests that, in the Spanish context—characterized by notable labor market precarity (Castro-Martín and Martín-García, 2016; Bueno and Brinton, 2019)—objective financial constraints may serve as more immediate barriers to family formation.

In Finland, while household net income is relevant, their role appears secondary to other strong predictors. For Finnish men this variable appears after initial splits related to partnership and, for a key group, partner’s employment. This position might suggest that while income matters, it operates within a context where other socio-relational factors also play a primary role (Golovina et al., 2024). Plausibly, Finland’s comprehensive welfare state might buffer some acute financial pressures, allowing non-financial considerations to surface more quickly in the decision-making hierarchy, though income thresholds evidently still matter for certain groups.

Germany emphasizes the couple’s joint educational capital—a proxy for long-term earning potential—over direct income measures. For both men and women, a partner’s tertiary education is strongly linked to positive intentions. This emphasis on “dual status” couples suggests that long-term earning potential and perceived stability, indexed by high educational attainment for both partners, might be more salient in Germany than immediate household income (Kreyenfeld and Konietzka, 2017; Kreyenfeld et al., 2023). Theoretically, in a system with robust baseline social security like Germany’s, the assurance of future financial stability, represented by human capital, could become a more critical differentiator than current income.

#### 5.1.3 Caregiving co-responsibility: varying thresholds of equity and the persistent gendered burden

The negotiation of caregiving is a critical area where gender-based needs become visible. This process often centers on the concept of the “second shift” (Hochschild and Machung, 1989), which refers to the domestic labor and care work that typically falls on women after their paid workday. Such negotiations reveal what McDowell et al. (2005) call “moral economies” of care. These are not financial systems, but rather the shared norms and expectations that define what is considered a fair or proper division of responsibilities in each society. How couples manage this second shift exposes these underlying rules. Fertility decisions often reflect a response to the perceived fairness of the domestic gender contract. Notably, the thresholds for what is considered an acceptable division of care vary significantly across Finland, Germany, and Spain, highlighting the existence of different moral economies.

In Finland, a substantial share of care undertaken by women (between 22 and 81% of tasks) is compatible with fertility intentions, suggesting an ideal of “managed dual participation.” Theoretically, the high value on gender equality in the Scandinavian regime likely sensitizes women to imbalances, making reasonably equitable care a prerequisite for further children, as an unsustainable “second shift” would conflict with career and well-being aspirations (Salmi and Lammi-Taskula, 2014; Eydal et al., 2018). For Finnish men, a specific node showed slightly higher intentions with lower male care involvement (within an egalitarian subgroup), a nuanced finding; however, the overall most favorable male profile included higher care involvement.

The German case reflects the tensions of a conservative-corporatist model in transition (Kreyenfeld and Konietzka, 2017). For cohabiting women not working full-time, fertility intentions are higher when they perceive their care involvement to exceed 45%. This threshold, approaching the equity point (50%), suggests that achieving a better-than-average balance is a positive factor. While the German system may facilitate combining work (often part-time for mothers) and family, the perceived fairness of the resulting care arrangement is crucial for further fertility (Trappe et al., 2015). It may not be about achieving perfect 50/50 equality, but whether the existing, often gendered, division is perceived as manageable and fair by the woman. For German men, higher educational capital correlates with both higher fertility intentions and a perception of greater equity in childcare (a male involvement between 30 and 50%).

In Spain, representing a regime with traditional gender roles (Sevilla-Sanz et al., 2010; Morero-Mínguez and Ortega-Gaspar, 2022), intentions for married women are higher if they perceive performing more than 35% of care tasks. A female involvement below this threshold is negatively associated with intentions, suggesting that a significant deviation from the primary caregiver role is not fully integrated into family formation expectations. For Spanish men, index care interacting with personal values for those without a work contract suggests a subtle renegotiation of male roles, perhaps more as an adaptive strategy in precarious situations than a widespread normative shift toward co-responsibility (Cukrowska-Torzewska and Matysiak, 2020). Deeply embedded cultural norms of familism and traditional gender roles in the Mediterranean regime likely mean that deviations toward more equitable care are still being negotiated and not yet institutionalized as in other regimes.

#### 5.1.4 Family and gender attitudes

The influence of individual values is modulated by each regime’s context. Our findings suggest values interact with structural conditions and normative expectations, sometimes leading to outcomes challenging straightforward assumptions about progressivism and fertility. In Finland, conservative values are associated with the highest fertility intentions among a subgroup of unemployed, lower-income women. This may reflect an “equality dilemma” (Oláh and Bernhardt, 2008), where women with high professional aspirations and egalitarian values perceive greater conflict between motherhood and professional goals, despite state support, leading to postponement or foregoing childbearing.

In Germany, structural factors like cohabitation and education often appear more prominent than family and gender attitudes. However, values can differentiate intentions within specific subgroups; for instance, non-cohabiting, tertiary-educated men with conservative to moderately progressive values show higher intentions than their more progressive counterparts. This might suggest that in the German context, often described by “egalitarian essentialism” (Lappegård et al., 2021), practical arrangements and perceived fairness of household gender roles may carry more immediate weight in reproductive decisions than abstract ideological stances for many. However, for certain subgroups, like these highly educated, non-cohabiting men, traditional values may still sustain fatherhood aspirations outside a stable partnership. The overall pattern suggests values matter, but their influence is perhaps more filtered through, or secondary to, certain socioeconomic and relational preconditions.

Spain, by contrast, reveals a nuanced interplay between traditional value markers and emerging family pathways. Marriage continues to hold symbolic and cultural significance, aligning with expectations for a Mediterranean regime where traditional institutions often retain considerable weight. However, our findings indicate that non-married women—particularly those who are economically independent or cohabiting—display the highest fertility intentions in the model, pointing to a diversification of pathways to motherhood (Domínguez-Folgueras and Castro-Martín, 2013). Economic autonomy and relationship stability outside of marriage thus appear to offer increasingly legitimate bases for family formation. Among Spanish men without a stable work contract, the interaction between caregiving responsibilities and personal values suggests that traditional orientations may continue to sustain fatherhood aspirations even in contexts of employment insecurity (Moreno and Crespi, 2017). The prominence of these traditional markers in Spain, compared to Finland’s more complex value landscape, may reflect a context where secularization and shifts toward individualized, progressive values are less uniformly advanced or encounter stronger countercurrents from established cultural family and gender norms.

### 5.2 Synthesizing cross-cutting themes: potential regime evolution, internal tensions, and theoretical reconsiderations

Synthesizing these findings, we move beyond a simple description of regime evolution. Instead, we interpret these patterns through the lens of the mobilization potential of gender-based needs. The internal tensions, hybridizations, and transitional dynamics we observe are not merely academic puzzles. They represent the macro-level outcomes of countless individual negotiations and acts of resistance. When personal aspirations for family and career clash with structural barriers, the resulting decisions—to postpone, to partner differently, to demand more from a partner—can drive social change.

Our dimensional analysis shows that contemporary European gender regimes are neither static nor monolithic. The comparative findings highlight their dynamic nature, marked by processes of potential regime evolution, significant internal tensions, and instances of “hybridization” or “misalignment” between theoretical models and real-life experiences (Pfau-Effinger, 2017). These cross-cutting themes challenge simplistic applications of regime typologies and call for a more nuanced understanding of how social, economic, and cultural forces interact to shape reproductive landscapes.

A prominent cross-cutting theme is the potential regime evolution suggested by patterns in Spanish data. While retaining historical Mediterranean imprints, the Spanish case shows indicators interpretable as signs of transition. The growing importance of female employment and economic independence as enablers of fertility intentions (even outside marriage), increasing viability of cohabitation, and emerging, albeit conditional, relevance of male care involvement might collectively point toward a departure from older norms. This “transitional dynamic” (Oláh et al., 2018; Bueno, 2020), if occurring, is not necessarily a linear progression but a complex reconfiguration creating its own challenges, particularly within a “stalled gender revolution” where structural supports for new family models may lag evolving aspirations. Exploring Spain as a potential regime in transition seems a fruitful avenue for future research, though this study’s limitations prevent definitive conclusions. Policies for a static, traditional model might be less effective if such a transition is underway.

A second key theme involves internal tensions and “paradoxical outcomes” even within regimes considered more stable or advanced in gender equality, like Finland. The unexpected association of conservative values with high fertility intentions among vulnerable women, or persistent relevance of male income, suggests the Scandinavian model’s lived experience is more heterogeneous than often portrayed. These findings may reflect an “equality dilemma” (Oláh and Bernhardt, 2008), where pursuing egalitarian ideals can generate new conflicts or highlight unresolved issues in reconciling work, family, and personal fullfilment for different subgroups. This complexity enriches the Nordic model, revealing areas where policy and societal norms may not be fully aligned or where diverse individual strategies emerge.

Similarly, the German case illustrates “egalitarian essentialism” (Lappegård et al., 2021), highlighting how policies for choice and work-family balance can coexist with, and sometimes inadvertently reinforce, underlying gendered care assumptions. The emphasis on couples’ joint educational capital and perceived care fairness, rather than solely individual employment/income for certain groups, points to a specific “German model” of navigating fertility decisions. This underscores how institutional frameworks can create unique pathways blending progressive goals with persistent traditional elements, leading to distinct hybridization.

Taken together, these observations call for a reconsideration of how rigid welfare and gender regime typologies are used. The coexistence of elements from different “ideal types” (hybridization) and the lack of alignment between policies, cultural values, and individual behaviors (misalignment) point to the need for a more flexible and context-sensitive approach. Instead of focusing solely on categorizing countries, future research should explore the pathways and factors that drive change, create internal variation, and shape individual agency within these broader frameworks. The divergent trajectories and internal complexities we have identified are valuable because they expand theoretical boundaries and highlight the ongoing, multifaceted renegotiation of gender relations and family life across Europe (Kwan and Choi, 2023).

These theoretical reconsiderations become even more pressing when viewed against recent demographic trends shown in Figure 1. The near convergence of actual fertility rates across our three countries in the last decade—particularly Finland’s steep decline since 2010 to levels similar to Germany and Spain by 2022—challenges traditional McDonald-esque welfare regime explanations for fertility patterns. This convergence suggests that traditional welfare and gender regime theories may be insufficient to explain contemporary reproductive dynamics.

Recent theoretical developments provide compelling frameworks for understanding this convergence. Vignoli et al.’s (2020) Narrative Framework emphasizes how fertility decisions are made under fundamental uncertainty, where individuals construct “narratives of the future” combining expectations, imaginaries, and structural constraints. Contemporary global uncertainties—economic volatility, climate change, and technological disruption—may be creating shared conditions that transcend national institutional arrangements. Similarly, Gortfelder et al.’s (2024) research on trust as a coping mechanism reveals how institutional trust functions as a resilience factor against uncertainty, suggesting that demographic convergence may reflect broader erosion of trust-based coping mechanisms across European societies. Our findings of internal tensions, hybridization, and paradoxical outcomes within regimes align with this demographic reality, suggesting that regime typologies’ explanatory power may be diminishing in the face of shared global pressures that overwhelm regime-specific institutional arrangements.

### 5.3 Limitations and future research

While this study provides valuable insights into the determinants of short-term fertility intentions, several limitations should be acknowledged. First, the cross-sectional nature of our data limits our ability to infer causal relationships between predictors and fertility intentions. Although we observe associations, longitudinal studies are needed to capture the dynamic nature of reproductive decision-making over time.

Second, although CART models offer a robust analytical framework for identifying key predictors and their interactions, they may oversimplify complex socio-cultural dynamics by focusing on decision thresholds. Future research could complement this quantitative approach with qualitative methodologies. For example, in-depth interviews or case studies, as suggested by Jurado et al. (2025), could capture nuanced experiences and subjective interpretations, providing a richer understanding beyond statistical segmentation.

Third, it is important to note the differential predictive capacity of our models across countries. Performance metrics indicate that the included variables demonstrate stronger predictive power for fertility intentions in Finland and Germany compared to Spain, particularly for Spanish women where their importance appears more moderate. This suggests other unmeasured factors might be especially salient in the Spanish context. Consequently, future research should prioritize exploring and validating alternative or additional predictors to better unravel the complexities shaping fertility intentions in Spain, potentially including more nuanced measures of economic precarity, uncertainty, social support networks, or specific cultural attitudes.

Fourth, the study’s focus on three specific countries, while enabling in-depth comparison across distinct gender regime types, naturally limits the generalizability of findings to other national contexts. Expanding the analysis to include a broader range of countries with varying levels of gender equality, welfare state provisions, and cultural backgrounds would provide a more comprehensive understanding of the interplay between macro-level gender regimes and micro-level fertility intentions. Particularly relevant for future comparative research would be situating the Spanish case alongside other Mediterranean regimes, such as Italy or Portugal (Aassve et al., 2024). As our discussion suggests, Spain exhibits characteristics indicative of a potential transition in its gender regime. A focused comparative analysis with countries sharing similar socio-cultural and institutional legacies could clarify whether patterns observed in Spain (e.g., the role of female employment, evolving cohabitation) are unique national developments or part of broader regional trends within Southern Europe.

Finally, the use of secondary data (GGS and INE surveys), while providing rich and harmonized information, inherently restricts the scope of variables available. These comprehensive surveys may not capture all potentially relevant dimensions influencing reproductive decision-making. Future research could benefit from primary data collection designed to incorporate a wider array of variables, such as more detailed attitudinal scales on gender roles, measures of social network influences, perceived institutional trust, or fine-grained indicators of cultural norms. Employing mixed-methods approaches in such primary data collection could also yield deeper insights.

## 6 Concluding remarks and policy implications

This comparative study of fertility intentions in Finland, Germany, and Spain, which set out to explore how contextual factors—in conjunction with individual and couple-level characteristics like employment, resources, caregiving distribution, and gender attitudes—shape short-term fertility intentions, reveals a complex interplay of socioeconomic, relational, and value-based factors. By developing predictive models using CART based on quantitative analysis of data from the GGS and the Spanish Fertility Survey, we examined the extent to which these individual and partner factors contribute to variations in intentions and how these determinants differ between men and women across distinct gender regimes. Our findings highlight that while broad regime typologies are useful, there is significant internal heterogeneity, potential regime evolution (notably in Spain), and nuanced interactions challenging simplistic interpretations. Beyond a demographic analysis, our findings lend themselves to an interpretation that views short-term fertility intentions as a useful indicator of underlying social pressures, potentially reflecting a latent mobilization in response to unmet gender-based needs.

A key policy implication, therefore, is addressing unequal care distribution, a critical factor for women across all contexts. Policies should promote genuine paternal co-responsibility beyond just parental leaves, with well-compensated, non-transferable paternal leave quotas (Grunow and Evertsson, 2022) being essential. However, tailored approaches are needed. In Finland, despite egalitarian ideals, findings suggest that while promoting male care is vital, managing women’s perceived overall care burden to prevent a deterring “second shift” is equally crucial. In Germany, where perceived care fairness was key for some women’s fertility intentions, policies should dismantle “egalitarian essentialism,” ensuring flexible work supports women’s careers and partners’ domestic participation, fostering a fair “gender contract.” For Spain, where high care burden significantly impacted married women, enhanced parental leave should be complemented by public campaigns to challenge cultural resistance, normalize shared care, and reframe fatherhood, addressing cultural lag, especially in traditional contexts. Viewing these issues through the lens of gender-based needs suggests that such policies are not just socially desirable, but may also address a significant source of tension influencing family life decisions.

Enhancing economic stability is also paramount, with policy levers differing by national context. In Spain, where employment status was salient for men’s and non-married women’s short-term fertility intentions, measures like improved job security, fair wages, and family financial support are vital. Active labor market policies addressing youth unemployment and precarious work can support male provider roles and female economic independence. A counterintuitive finding for some lower-income Spanish men suggests non-economic factors also merit policy reflection. Even in Finland, men’s household income remained key for intentions; policies should ensure economic security, including affordable housing. In Germany, where couples’ combined educational capital was significant, policies should facilitate dual high-skill career paths and ensure education translates to secure employment for both. This suggests that economic precarity may function as more than a simple constraint; it can be interpreted as a structural barrier that conflicts with the fulfillment of life projects, potentially generating widespread, albeit quiet, social pressures.

Achieving a comprehensive “gender revolution” requires synergistic progress in public and private spheres. Foundational structural policies promoting equality in employment and care must be complemented by efforts addressing individual attitudes. The complex, sometimes paradoxical role of attitudes (e.g., conservative attitudes linked to higher short-term fertility intentions for some vulnerable Finnish women and specifically German men) means structural policies should be accompanied by efforts to challenge restrictive gender norms and foster egalitarian attitudes—through awareness campaigns and education—while acknowledging value diversity and avoiding one-size-fits-all approaches (Grunow and Evertsson, 2022). This complexity might indicate that if we consider these choices as a form of individual-level mobilization, it is not a monolithic process, but a fragmented one where different group appear to leverage different resources to navigate their life course.

Policy implications must consider study limitations (cross-sectional nature, specific variables) and realistic expectations. While policies can foster equality, work-life balance, and individual fertility desires, their capacity to reverse aggregate fertility declines, especially in low-fertility contexts, may be limited (Gauthier and Gietel-Basten, 2024). In conclusion, this research underscores the need for context-specific, gender-transformative approaches to fertility intentions. It suggests that policymakers could benefit from creating comprehensive, enabling environments that are responsive not only to demographic trends, but also to the underlying social tensions our findings hint at. By considering fertility intentions through this lens, we can better appreciate how the private decision to form a family may also be understood as a profoundly public matter, reflecting the ongoing negotiation of gender-based needs in contemporary Europe. Future longitudinal and mixed-methods research is essential to further unravel these complexities.

## Data Availability

The datasets presented in this study can be found in online repositories. The names of the repository/repositories and accession number(s) can be found in the article/Supplementary material.

## Footnotes

^1^The authors acknowledge the significant disparity in sample sizes between the countries. However, as the CART models were constructed independently for each national subsample, this difference does not affect the internal validity of each country-specific model. The Finnish sample (*N* = 2,229) is sufficiently large to ensure the robustness of the recursive partitioning analysis. The primary potential impact of a smaller sample size would be a more parsimonious tree structure, which is an inherent feature of the CART methodology to prevent overfitting, rather than a limitation that compromises the comparative findings.

^2^The threshold used to differentiate these value orientations was an index values score ≤ 0.79 for the conservative to moderately progressive group.
